# Central Role of Sibling Small RNAs NgncR_162 and NgncR_163 in Main Metabolic Pathways of Neisseria gonorrhoeae

**DOI:** 10.1128/mbio.03093-22

**Published:** 2023-01-04

**Authors:** Thomas Steiner, Marie Zachary, Susanne Bauer, Martin J. Müller, Markus Krischke, Sandra Radziej, Maximilian Klepsch, Bruno Huettel, Wolfgang Eisenreich, Thomas Rudel, Dagmar Beier

**Affiliations:** a Chair of Microbiology, Theodor-Boveri-Institute, University of Würzburg, Würzburg, Germany; b Bavarian NMR Center–Structural Membrane Biochemistry, Department of Chemistry, Technical University of Munich, Garching, Germany; c Department of Pharmaceutical Biology, Julius von Sachs Institute, University of Würzburg, Würzburg, Germany; d Max Planck Genome Centre, Cologne, Germany; e Helmholtz Institute for RNA-Based Infection Research (HIRI), Helmholtz Center for Infection Research (HZI), Würzburg, Germany; National Institute of Child Health and Human Development (NICHD)

**Keywords:** sRNA, *Neisseria gonorrhoeae*, posttranscriptional regulation, amino acid transporter, bipartite metabolism

## Abstract

Small bacterial regulatory RNAs (sRNAs) have been implicated in the regulation of numerous metabolic pathways. In most of these studies, sRNA-dependent regulation of mRNAs or proteins of enzymes in metabolic pathways has been predicted to affect the metabolism of these bacteria. However, only in a very few cases has the role in metabolism been demonstrated. Here, we performed a combined transcriptome and metabolome analysis to define the regulon of the sibling sRNAs NgncR_162 and NgncR_163 (NgncR_162/163) and their impact on the metabolism of Neisseria gonorrhoeae. These sRNAs have been reported to control genes of the citric acid and methylcitric acid cycles by posttranscriptional negative regulation. By transcriptome analysis, we now expand the NgncR_162/163 regulon by several new members and provide evidence that the sibling sRNAs act as both negative and positive regulators of target gene expression. Newly identified NgncR_162/163 targets are mostly involved in transport processes, especially in the uptake of glycine, phenylalanine, and branched-chain amino acids. NgncR_162/163 also play key roles in the control of serine-glycine metabolism and, hence, probably affect biosyntheses of nucleotides, vitamins, and other amino acids via the supply of one-carbon (C_1_) units. Indeed, these roles were confirmed by metabolomics and metabolic flux analysis, which revealed a bipartite metabolic network with glucose degradation for the supply of anabolic pathways and the usage of amino acids via the citric acid cycle for energy metabolism. Thus, by combined deep RNA sequencing (RNA-seq) and metabolomics, we significantly extended the regulon of NgncR_162/163 and demonstrated the role of NgncR_162/163 in the regulation of central metabolic pathways of the gonococcus.

## INTRODUCTION

Neisseria gonorrhoeae is the causative agent of the disease gonorrhea, which is one of the most common sexually transmitted infections. The World Health Organization estimates 106 million new cases each year (WHO, 2012). However, alarmingly, not only are the numbers of infections rising but also multidrug-resistant gonococcal strains are emerging increasingly, raising the threat of untreatable gonorrhea (1). Gonococci mostly infect the mucosa of the urogenital tract, causing urethritis in men and cervicitis in women, but they also infect the pharynx, rectum, and conjunctiva (2). In rare cases, they can enter the bloodstream, causing systemic disseminated gonococcal disease with manifestations like endocarditis, arthritis, dermatitis, and sepsis (1).

The ability to infect different tissues requires effective regulatory mechanisms to ensure the pathogen’s rapid adaptation to changing environments. Noncoding RNAs have been recognized as an important class of posttranscriptional regulators of bacterial metabolism and virulence (3, 4). *Trans*-acting small RNAs (sRNAs) are typically transcribed from intergenic regions and undergo short, imperfect base-pairing interactions with their target mRNAs. Most frequently, sRNA binding to the 5′ untranslated region (UTR) of the target mRNA affects translation initiation by either obstructing the ribosome binding site (RBS) or by unfolding an intrinsic inhibitory secondary structure of the mRNA (5, 6). More recently, it was shown that negative regulation at the 5′ UTR affecting translation initiation can also be accomplished by sRNA binding to ribosome standby sites or translational enhancer elements (7–9). Inhibition of translation is usually accompanied by increased ribonucleolytic target mRNA degradation in the absence of translating ribosomes. However, sRNA binding to both the 5′ UTR and the coding region can also directly impact the mRNA half-life by either prevention of mRNA decay or stimulation of mRNA turnover by the active recruitment of RNases (5, 10). Yet another mode of action of sRNAs is the inhibition of Rho-dependent termination by blocking rut-sites, which were found to be frequently located in unusually long 5′ UTRs (11). Most studied sRNAs from Gram-negative bacteria require an interaction with an RNA chaperone like Hfq or ProQ in order to exert their function (12, 13).

Interestingly, multicopy sRNAs exhibiting a high degree of sequence identity, termed “sibling sRNAs,” were identified in some bacteria. Given their similarity, these sibling sRNAs may share a seed region and therefore would be expected to act redundantly on the same target mRNAs. However, they may yet exert unique functions due to differential expression of sRNA-encoding genes in response to particular environmental stimuli by targeting different mRNAs via sequence motifs which are not conserved between the siblings or by mechanisms of gene regulation unique to each sibling (reviewed in reference 14). The largest group of multicopy sRNAs known so far is represented by the LhrC family of Listeria monocytogenes, which comprises the highly homologous sRNAs LhrC1to LhrC5 as well as Rli22 and Rli33-1, which exhibit a lower degree of sequence conservation (15, 16). LhrC family sRNAs have an additive effect in target regulation, while the regulatory effect of a single sRNA seems to be rather subtle (16). The regulation of unique targets by sibling sRNAs is exemplified by the AbcR sRNAs of Sinorhizobium meliloti (17, 18).

A pair of sibling sRNAs has also been described in N. gonorrhoeae and Neisseria meningitidis (19–21). These sRNAs, termed NgncR_162 and NgncR_163 (NgncR_162/163) in N. gonorrhoeae, are encoded adjacent to each other, exhibit 78% sequence identity, and fold into a similar secondary structure comprising three stem-loops (SLs). They are strongly expressed under standard laboratory growth conditions, but NgncR_163 is considerably more abundant than NgncR_162 (19). The *Neisseria* sibling sRNAs were shown to regulate the expression of genes involved in transcription regulation, amino acid uptake, and basic metabolic processes, including the methylcitric acid and citric acid (tricarboxylic acid [TCA]) cycles. All previously identified target genes are negatively regulated via hybridization of the sRNAs to the RBS of the target mRNA (19–21). Complete functional redundancy of the sibling sRNAs has been demonstrated experimentally (19–21) for a subset of targets.

To analyze the role of the sibling sRNAs in the neisserial metabolism in further detail, we combined transcriptome analysis and the investigation of carbon fluxes based on metabolomics and stable isotope incorporation experiments. By this integrative approach, several new target genes, including positively regulated targets, were identified and the sibling sRNAs were shown to interfere with sugar catabolism, the TCA cycle, serine-glycine metabolism, and the transport of glycine, phenylalanine, and branched-chain amino acids (BCAA).

## RESULTS

### Global transcriptome analysis reveals new members of the NgncR_162/163 regulon.

**FIG 1:**
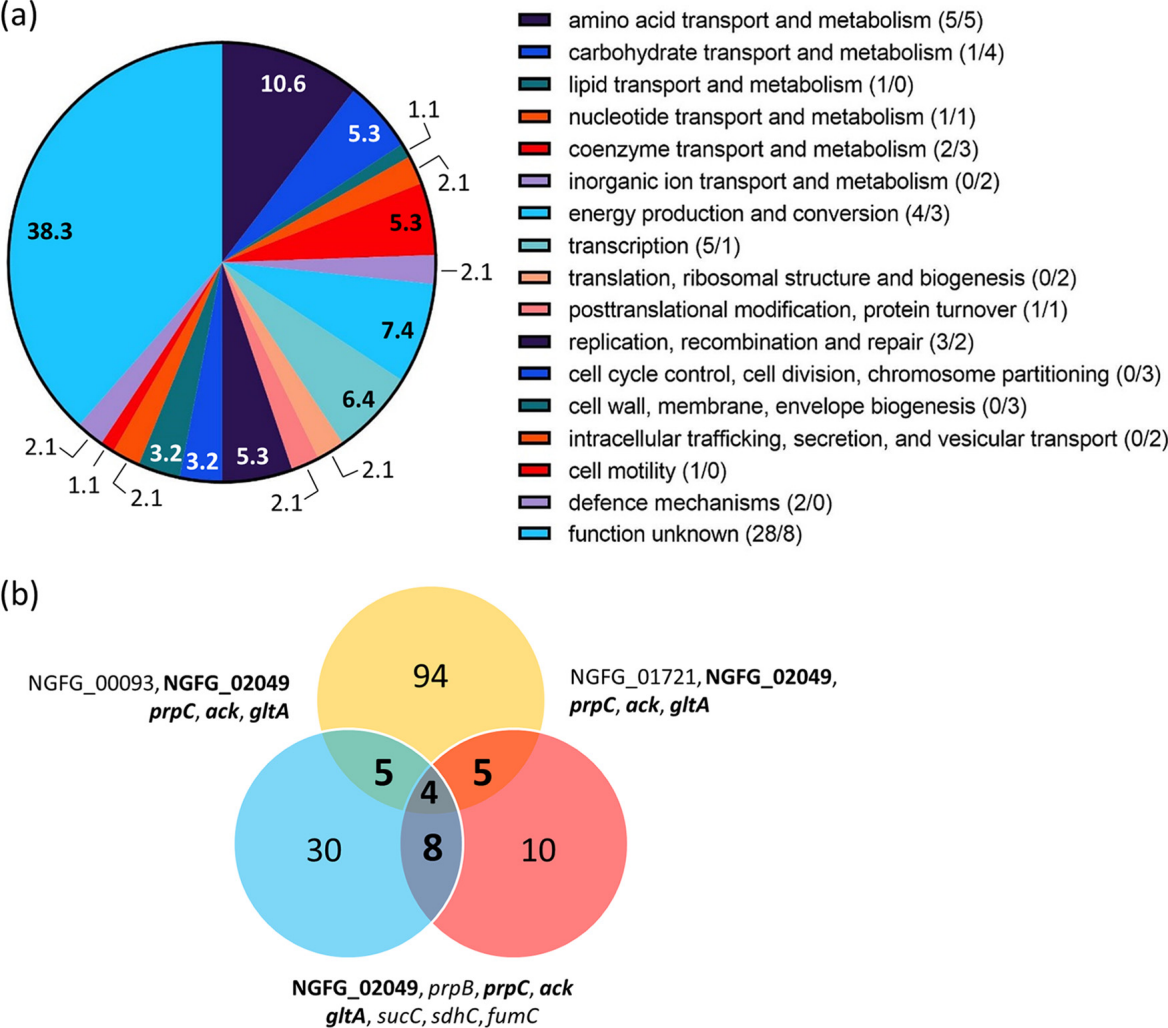
Graphical representation of transcriptomics data presented in Table 1. (a) Pie chart depicting the biological role of putative protein-encoding sibling sRNA target genes identified by RNA-seq. Numbers within or next to the segments of the graph represent the percentages of putative sibling sRNA targets (*n* = 94) belonging to the respective functional categories listed on the right. Numbers given in parentheses in the pie chart legend represent the numbers of putative targets from the respective categories which are repressed or activated by the sibling sRNAs (repressed, *n* = 54/activated, *n* = 40). (b) Venn diagram representing intersecting sets of *Neisseria* sibling sRNA target genes identified in this study (yellow) and in the works of Pannekoek et al. (21) (blue) and Bauer et al. (19) (red). Pannekoek et al. investigated the sibling sRNA regulon in N. meningitidis; for a better comparison, N. gonorrhoeae orthologs of the N. meningitidis targets are listed in the figure. Numbers in the diagram represent the number of targets identified in the respective study, as well as the number of common targets which are also listed next to overlapping segments. Common targets of the three studies are highlighted in bold.

We and others previously identified target genes of the sibling sRNAs NgncR_162/163 and their homologues in N. meningitidis by *in silico* analysis of the sRNA-mRNA interaction (19, 20) and comparative mass spectrometric analysis of cell lysates from wild-type bacteria and an sRNA double deletion mutant (21). To obtain more complete insight into the NgncR_162/163 regulon, we now compared the gene expression profiles of N. gonorrhoeae wild-type MS11 and the sRNA ΔΔ162/163 double deletion mutant (19) grown in rich proteose peptone medium (PPM) by deep RNA sequencing (RNA-seq). Genes differentially expressed in the two strains were assessed using DESeq2 (22). A number of the 94 protein coding genes (54 upregulated, 40 downregulated) exhibited expression ratios below 0.75 and above 1.5 (adjusted *P* value [*q*] of <0.05), which was considered differentially expressed (Table 1). Differentially expressed genes mostly belonged to the following functional categories (COG): amino acid transport and metabolism (*n* = 10), energy production and conversion (*n* = 7), transcription (*n* = 6), and unknown function (*n* = 36) (Fig. 1a). Putatively regulated open reading frames (ORFs) encoding hypothetical proteins of unknown function tend to be very short and are most frequently located in the N. gonorrhoeae MS11 homologues of the *maf* genomic islands of N. meningitidis encoding secreted polymorphic toxins (*mafB*), their specific immunity proteins (*mafI*), and alternative MafB C-terminal domains (MafB-CT) or MafI modules (23). However, functional enrichment analysis using a hypergeometric test did not indicate significant overrepresentation of these COG classes. From the NgncR_162/163 target genes validated previously by real-time quantitative PCR (qRT-PCR) (19), the NGFG_01721 and NGFG_02049 genes, *prpC*, *ack*, and *gltA* were detected by RNA-seq (Fig. 1b), whereas *prpB*, *gdhR*, *fumC*, and *sucC* did not meet the applied cutoff, reflecting differences in the sensitivity of the two RNA quantification techniques. Furthermore, seven putative N. gonorrhoeae noncoding RNAs (24) were found to be differentially transcribed in the ΔΔ162/163 mutant (Table 1).

**TABLE 1:** Genes differentially expressed in MS11 ΔΔ162/163 within a <0.75 and >1.5 cutoff for fold expression change (ratio) compared to MS11 (*q* < 0.05)^*a*^

Gene	*q* value	Ratio	Annotation	COG category
NGFG_01721*	1.05E–62	6	Sodium alanine symporter	Amino acid transport and metabolism
NGFG_01722*	2.34E–35	3.2266	d-Amino acid dehydrogenase (*dadA*)	Amino acid transport and metabolism
NGFG_00249*	2.76E–12	2.9282	Citrate transporter	Energy production and conversion
NGFG_02415	6.78E–08	2.514	Hypothetical protein (maf)	Function unknown
NGFG_01514	2.48E–06	2.2191	Glycine cleavage system H protein (*gcvH*)	Amino acid transport and metabolism
NGFG_02343	9.32E–07	2.1585	Hypothetical protein (maf)	Function unknown
NGFG_02102	7.26E–07	2.1435	Phage protein	Transcription
NGFG_02342	5.91E–04	1.9494	Hypothetical protein (maf)	Function unknown
NGFG_02349	1.53E–04	1.9319	Hypothetical protein (maf)	Function unknown
NGFG_00366	0.0011	1.9265	Hypothetical protein (maf)	Function unknown
NGFG_00721	6.95E–05	1.9212	Phage protein	Function unknown
NGFG_01411*	4.77E–06	1.9079	Acetate kinase (*ack*)	Nucleotide transport and metabolism
NGFG_02170	1.54E–07	1.8751	Lrp/AsnC family transcriptional regulator	Transcription
NGFG_01937*	1.39E–05	1.8699	Amino acid/peptide transporter	Amino acid transport and metabolism
NGFG_01163*	0.0105	1.8635	Iron-sulfur cluster assembly transcription factor IscR	Transcription
NGFG_00814*	1.39E–05	1.8596	Citrate synthase (*gltA*)	Carbohydrate transport and metabolism
NGFG_00720	9.71E–04	1.8378	Phage repressor protein	Transcription
NGFG_01491	0.0017	1.8365	Hypothetical protein	Function unknown
NGFG_00699	0.0021	1.8239	Hypothetical protein	Function unknown
NGFG_00952	0.0012	1.8201	Single-strand DNA-binding protein	Replication, recombination, and repair
NGFG_01349	0.0064	1.8075	Hypothetical protein (maf)	Function unknown
NGFG_02049*/ NGFG_02050	0.0037/ 0.0312	1.8075/ 1.6178	3-Hydroxyisobutyrate dehydrogenase	Lipid transport and metabolism
NGFG_00480	2.45E–04	1.795	Thioredoxin	Posttranslational modification, protein turnover
NGFG_00981	0.01	1.7888	Conjugative transfer pilus assembly protein TraH	Function unknown
NGFG_02348	4.23E–04	1.7851	Hypothetical protein (maf)	Function unknown
NGFG_00447	0.0118	1.7839	Hypothetical protein	Function unknown
NGFG_00507	1.25E–04	1.774	Hypothetical protein	Defense mechanisms
NGFG_01311	0.0247	1.7605	Hypothetical protein	Function unknown
NGFG_02500	0.005	1.7569	Mobilization protein	Function unknown
NGFG_02499	0.0105	1.7171	Relaxase/mobilization protein	Function unknown
NGFG_01842	0.0064	1.7088	Thiamine biosynthesis protein ThiC	Coenzyme transport and metabolism
NGFG_02237	0.0363	1.7076	Opacity protein	Function unknown
NGFG_00670	0.019	1.7041	Hypothetical protein (maf)	Function unknown
NGFG_01536	0.0076	1.6876	Hypothetical protein (maf)	Function unknown
NGFG_02345	0.0064	1.6853	Hypothetical protein (maf)	Function unknown
NGFG_00448	0.0035	1.6806	Restriction endonuclease	Defense mechanisms
NGFG_00971	0.0042	1.6806	Hypothetical protein	Function unknown
NGFG_01821	0.0438	1.6609	Pilin PilE	Cell motility
NGFG_00953	0.0191	1.6598	DNA topoisomerase III	Replication, recombination, and repair
NGFG_00093	3.74E–07	1.6369	d-Methionine ABC transporter substrate-binding protein	Amino acid transport and metabolism
NGFG_01303	2.06E–04	1.6358	Phage protein	Transcription
NGFG_02463	0.0089	1.629	Hypothetical protein	Function unknown
NGFG_02363	0.0453	1.6234	Hypothetical protein	Function unknown
NGFG_02204	0.017	1.6166	Hypothetical protein (maf)	Function unknown
NGFG_00671	0.0217	1.6044	Hypothetical protein (maf)	Function unknown
NGFG_02439	0.0371	1.5966	Hypothetical protein (maf)	Function unknown
NGFG_01404*	0.0053	1.59	2-Methylcitrate synthase (*prpC*)	Energy production and conversion
NGFG_01407*	0.0056	1.59	Aconitate hydratase (*acn*)	Energy production and conversion
NGFG_02209	0.0402	1.5834	Hypothetical protein	Function unknown
NGFG_00343	0.0133	1.5551	Anhydrase	Carbohydrate transport and metabolism
NGFG_01898	0.0175	1.5443	rRNA small subunit methyltransferase I	Coenzyme transport and metabolism
NGFG_02247	0.0216	1.5379	Endonuclease	Replication, recombination, and repair
NGFG_00963	0.0442	1.5326	Hypothetical protein	Function unknown
NGFG_02205	0.0128	1.5011	Hypothetical protein (maf)	Function unknown
NGFG_00045*	1.33E–27	0.2552	NSS family neurotransmitter Na^+^ symporter	Function unknown
NGFG_02042	2.40E–10	0.4796	Acetolactate synthase large subunit (*ilvB*)	Amino acid transport and metabolism
NGFG_01146*	3.72E–05	0.5126	Iron-sulfur cluster repair protein (*dnrN*)	Cell cycle control, cell division, chromosome partitioning
NGFG_02041	1.69E–09	0.5385	Acetolactate synthase small subunit (*ilvH*)	Amino acid transport and metabolism
NGFG_01353	0.0161	0.5735	PiT family inorganic phosphate transporter	Inorganic ion transport and metabolism
NGFG_02153	1.37E–07	0.5897	Nitric oxide reductase subunit B (*norB*)	Inorganic ion transport and metabolism
NGFG_02056	0.0113	0.6143	4-Carboxymuconolactone decarboxylase	Function unknown
NGFG_00825	0.0256	0.6194	Oxidoreductase	Energy production and conversion
NGFG_02039	1.67E–05	0.6268	Ketol-acid reductoisomerase (*ilvC*)	Coenzyme transport and metabolism
NGFG_02040	0.0017	0.63	Hypothetical protein	Function unknown
NGFG_00254	5.91E–04	0.6346	Protein-export protein SecB	Intracellular trafficking, secretion, and vesicular transport
NGFG_02065	0.0026	0.6426	2.3-Bisphosphoglycerate-dependent phosphoglycerate mutase	Carbohydrate transport and metabolism
NGFG_02154*	0.0113	0.6457	Copper-containing nitrite reductase *(aniA)*	Energy production and conversion
NGFG_01516*	0.0081	0.6471	Flavin mononucleotide-binding protein (NosR/NirI family protein)	Transcription
NGFG_01727	0.0123	0.6498	Cell division topological specificity factor MinE	Cell cycle control, cell division, chromosome partitioning
NGFG_02044	1.58E–04	0.6502	ATP phosphoribosyltransferase	Nucleotide transport and metabolism
NGFG_01710	0.0011	0.6511	Ribosome-recycling factor	Translation, ribosomal structure, and biogenesis
NGFG_00252	5.24E–05	0.6597	RNase G	Translation, ribosomal structure, and biogenesis
NGFG_00360	0.0487	0.6602	Inorganic pyrophosphatase	Energy production and conversion
NGFG_00893	0.0137	0.6625	Hypothetical protein	Function unknown
NGFG_02111	0.0256	0.6657	Lactoylglutathione lyase (*gloA*)	Amino acid transport and metabolism
NGFG_00423	1.58E–04	0.6717	Rare lipoprotein B	Cell wall, membrane, envelope biogenesis
NGFG_00824	0.0031	0.6722	Hypothetical protein	Function unknown
NGFG_01728	0.0175	0.6737	Septum site-determining protein MinD	Cell cycle control, cell division, chromosome partitioning
NGFG_00779	0.0083	0.6792	Aspartate kinase	Amino acid transport and metabolism
NGFG_01544	8.13E–04	0.6816	M61 family glycyl aminopeptidase	Function unknown
NGFG_00765	0.0159	0.693	Ribose-5-phosphate isomerase A	Carbohydrate transport and metabolism
NGFG_01810	0.0017	0.695	UDP-glucose 4-epimerase	Cell wall, membrane, envelope biogenesis
NGFG_01564*	0.0087	0.7061	NSS family neurotransmitter Na^+^ symporter	Function unknown
NGFG_02259	0.0493	0.7076	Opacity protein	Cell wall, membrane, envelope biogenesis
NGFG_01941	0.0053	0.71	Oxidoreductase	Function unknown
NGFG_01323	0.0113	0.724	Phage integrase/recombinase	Replication, recombination and repair
NGFG_02263*	0.0452	0.7265	Glucose/galactose transporter	Carbohydrate transport and metabolism
NGFG_02419	0.0033	0.732	MafB family adhesin protein	Intracellular trafficking, secretion, and vesicular transport
NGFG_00052	0.0442	0.7351	*S*-Ribosylhomocysteine lyase	Coenzyme transport and metabolism
NGFG_02407	0.0068	0.7366	Phosphoserine aminotransferase	Amino acid transport and metabolism
NGFG_02066	0.0064	0.7371	DNA topoisomerase 4 subunit A	Replication, recombination, and repair
NGFG_01497	0.0271	0.7387	Heat shock protein GrpE	Posttranslational modification, protein turnover
NGFG_01354	0.0472	0.7443	Oxygen-independent coproporphyrinogen III oxidase	Coenzyme transport and metabolism
NGFG_00557	0.0134	0.7485	ADP-l-glycero-d-manno-heptose-6-epimerase	Carbohydrate transport and metabolism
NgncR_005	0.0419	0.7356	Noncoding RNA (115 nt, antisense to NGFG_00188)	
NgncR_136	0.0185	1.7963	Noncoding RNA (265 nt, partially antisense to NGFG_02092)	
NgncR_191	0.0041	1.9807	Noncoding RNA (896 nt, antisense to NGFG_02436)	
NgncR_199	0.0076	1.9132	Noncoding RNA (188 nt, intergenic)	
NgncR_201	2.06E−08	2.9282	Noncoding RNA (287 nt, intergenic)	
NgncR_210	0.0064	1.7231	Noncoding RNA (429 nt, intergenic)	
NgncR_250	2.45E−04	1.8816	Noncoding RNA (115 nt, intergenic)	

a RNA-seq was performed with samples from three biological replicates each. Note that the NGFG_02050 gene encodes the C-terminal part of 3-hydroxyisobutyrate dehydrogenase (NGFG_02049) due to a frameshift in the N. gonorrhoeae MS11 genome sequence (NC_022240.1). The designation “maf” in cases of ORFs annotated as hypothetical proteins indicates their location within N. gonorrhoeae MS11 homologs of the *maf* genomic islands of N. meningitidis (23). Classification according to COG categories (https://www.ncbi.nlm.nih.gov/research/cog) shown in column 5 was performed using EggNOG 5.0.0 (http://eggnog5.embl.de). Noncoding RNAs were annotated in the transcriptome analysis performed by Remmele et al. (24). NgncR162/163 target genes which were validated by qRT-PCR are highlighted by gray shading (reference 19 and this study). Genes which were deregulated in a Δ*hfq* mutant of N. meningitidis are marked with an asterisk (25).

**FIG 2:**
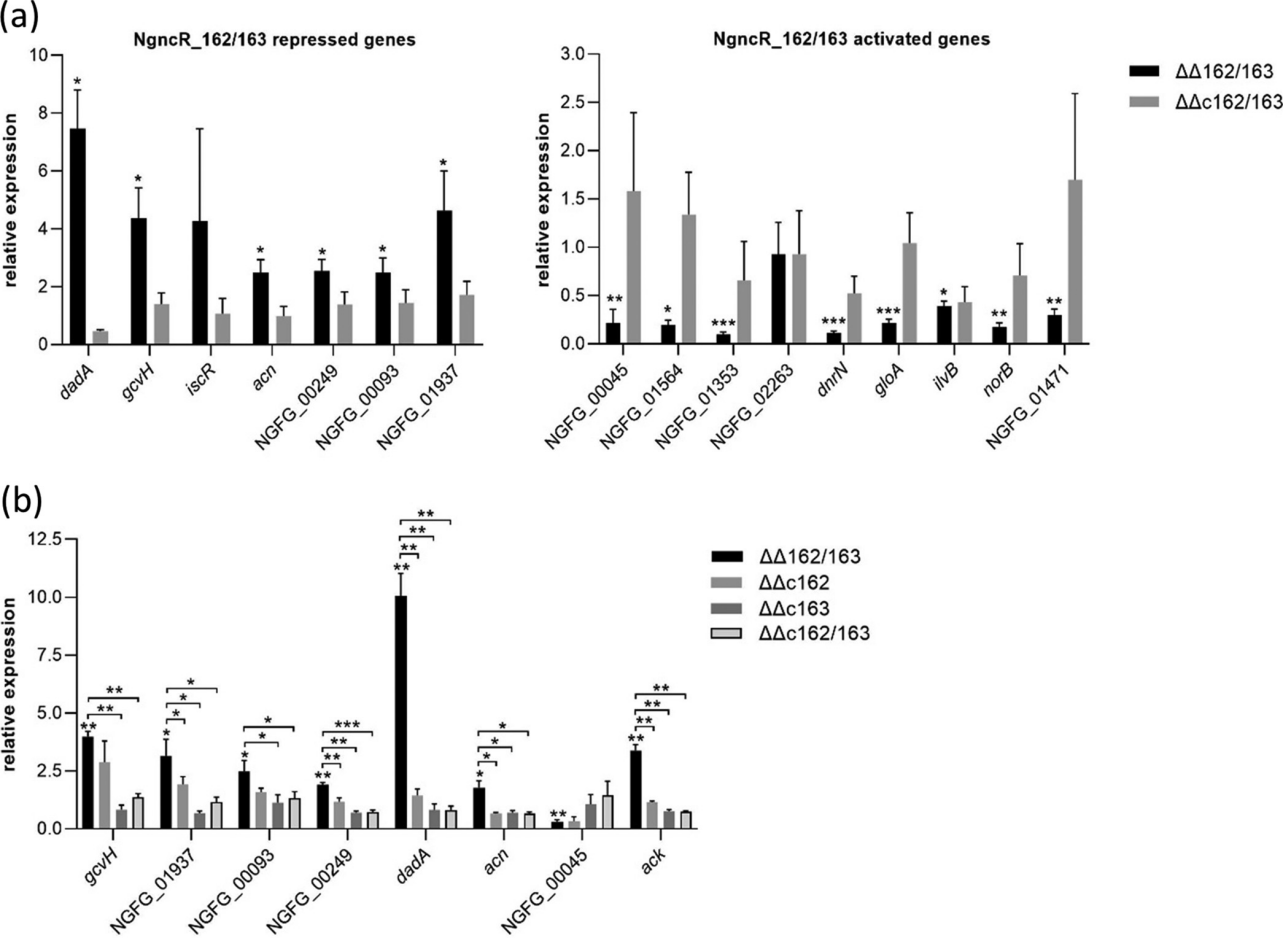
Validation of putative NgncR_162/163 target genes by qRT-PCR. (a) Transcript levels of selected genes which were found differentially expressed in the absence of the sibling sRNAs by RNA-seq were analyzed by qRT-PCR in strain MS11, the ΔΔ162/163 sRNA double deletion mutant, and the ΔΔc162/163 complemented strain cultivated in PPM. The ratios (fold change) of the transcript amount relative to that of the wild-type MS11 (mutant versus wild-type; wild-type normalized to 1) are depicted. (b) In the case of *gcvH*, NGFG_01937, NGFG_00093, NGFG_00249, *dadA*, *acn*, and NGFG_00045, transcript levels were also assessed in derivatives of MS11 ΔΔ162/163 complemented with either NgncR_162 (ΔΔc162) or NgncR_163 (ΔΔc163). *ack* was used as a control, since the sibling sRNAs were previously shown to display functional redundancy on this target (19). The indicated ratios represent the mean of the results of qRT-PCR experiments performed in triplicate using cDNAs obtained from at least three independent RNA preparations. Error bars indicate standard deviations. Statistical significance was determined using Student's *t* test analysis (*, *P* < 0.05; **, *P* < 0.01; ***, *P* < 0.001).

Fifteen protein coding putative NgncR_162/163 target genes with predicted metabolic functions were selected for target validation via qRT-PCR analysis (Fig. 2a) in the ΔΔ162/163 mutant and the complemented mutant carrying both sRNA genes integrated into the *iga*-*trpB* locus (ΔΔc162/163) (19). Differential expression in the ΔΔ162/163 mutant could be confirmed, except for that of the sugar transporter NGFG_02263. Gene *ilvB* (NGFG_02042) from the BCAA biosynthetic pathway was significantly downregulated in the ΔΔ162/163 mutant, but its expression did not revert in the ΔΔc162/163 complemented mutant. Validated target genes encode proteins putatively involved in the transport of amino acids or peptides (NGFG_00045 and NGFG_01564 [members of the neurotransmitter sodium symporter {NSS} family], NGFG_00093, and NGFG_01937), citrate (NGFG_00249), and inorganic phosphate (NGFG_01353), as well as NGFG_01722, whose gene is cotranscribed with the putative alanine symporter NGFG_01721 gene and encodes d-alanine dehydrogenase (*dadA*). Furthermore, differential expression of lactate permease (NGFG_01471) showing a ratio slightly above the applied cutoff in RNA-seq (0.765779; *q* = 0.0191) could be validated by qRT-PCR (Fig. 2a). Another validated target, aconitate hydratase (*acn*), belongs to the methylcitric acid cycle, which was previously shown to be controlled by the sibling sRNAs (19, 20). Interestingly, Gene *gcvH* (NGFG_01514) was found to be upregulated in the absence of the sibling sRNAs. GcvH is a component of the glycine cleavage system that converts glycine to CO_2_, NH_3_, and 5,10-methylene-tetrahydrofolate (5,10-MTHF), suggesting a role for the sibling sRNAs in purine, histidine, thymine, panthothenate, and methionine synthesis via control of the supply of C1 units. Furthermore, RNA-seq suggested a contribution of the sibling sRNAs in the control of iron-sulfur cluster synthesis and homeostasis (*iscR*, *dnrN*) and anaerobic respiration (*aniA*, *norB*, NGFG_01516).

Notably, 16 of the putative targets of the sibling sRNAs listed in Table 1 as well as lactate permease were also differentially expressed in an *hfq* deletion mutant of N. meningitidis (25). Since the sibling sRNAs were shown to bind Hfq in both N. meningitidis and N. gonorrhoeae and their stability was largely diminished in its absence (20; E. Heinrichs and T. Rudel, unpublished), deregulation of transcript levels in the *hfq* mutant is likely to be the consequence of posttranscriptional regulation mediated by the sibling sRNAs. In the case of NGFG_01471, differential expression in the ΔΔ162/163 mutant can be explained by an indirect effect, because lactate permease was shown to be regulated by GdhR (26), which itself is a target of the sibling sRNAs (19).

Transcription of three genes encoding putative noncoding RNAs (NgncR_199, NgncR_201, and NgncR_210) was also analyzed by qRT-PCR, but differential expression could not be validated using this method (data not shown).

To test the functional redundancy of NgncR_162 and NgncR_163 in the regulation of newly identified targets, we investigated as an example the impact of the individual sibling sRNAs as posttranscriptional regulators of *gcvH*, NGFG_01937, NGFG_00249, NGFG_00093, *acn*, *dadA*, and NGFG_00045. Complementation of the ΔΔ162/163 mutant with NgncR_163 restored mRNA abundance to wild-type levels, whereas complementation by NgncR_162 was less efficient for some of the investigated targets. Functional redundancy was observed only in the case of NGFG_00093, *acn*, and *dadA* target genes (Fig. 2b). These data suggest that the individual siblings act in a hierarchical manner on certain targets while exhibiting complete functional redundancy on others.

### sRNA-target interaction is predicted to occur within the 5′ UTR and coding region of the regulated mRNAs.

All targets identified previously (19–21) are negatively regulated by the sibling sRNAs via mRNA-sRNA duplex formation, resulting in obstruction of the RBS. The sRNA-mRNA interaction involved the single-stranded region of stem-loop 2 (SL2) and in some cases the single-stranded region connecting SL1 and SL2 (SSR1) (see Fig. S1a in the supplemental material). Complementarity between the validated new target mRNAs and the sibling sRNAs was analyzed using IntaRNA (27) (Fig. S1 and S2). In accordance with negative regulation of *dadA*, NGFG_00249, NGFG_01937, NGFG_00093, and *glyA* (see below), obstruction of the RBS by the SL2 loop or SSR1 sequence was predicted for both sRNAs (Fig. S1b). Surprisingly, also in the case of the positively regulated target *norB*, a region of complementarity to SL2 overlapping the RBS was detected. Furthermore, the 5′ UTR of *dnrA* shows complementarity to the SL2 sequence of both sRNAs, but in the case of NgncR_163, a different interaction site within the coding sequence (CDS) which engages the SL1 loop sequence was predicted. In the case of the other members of the NgncR_162/163 regulon, base-pairing interactions are likely to occur within the CDS (Fig. S2). IntaRNA analysis detected similar regions of complementarity between NGFG_00045, *iscR*, and *gloA* mRNAs and the SSR1 and SL2 sequences of both sRNAs. NgncR_162 exhibited extended complementarity with *gloA* mRNA, which also engages the single-stranded region connecting SL2 and SL3 (SSR2) and part of the SL3 sequence. In the case of *gcvH*, NGFG_01564, NGFG_01353, and *acn*, IntaRNA predictions differed for the two sRNA molecules. NGFG_01564 and *gcvH* mRNAs exhibited segments of 8 to 13 nucleotides (nt) with base-pairing capability to the SL2 sequence of both sRNAs or the SSR1 sequence of NgncR_162. The SL1 and SSR1 sequence of NgncR_162 showed partial complementarity to a 28-nt region within the CDS of NGFG_01353, while NgncR_163 was predicted to interact with a different region of NGFG_01353 via the SL2 and SSR2 sequence (which, however, is conserved in both sRNAs [Fig. S1a]). Within the *acn* CDS (1,920 bp), several regions of complementarity with the sibling sRNAs were detected (IntaRNA energy scores, −4.46 to −10.35 kcal/mol), one of which is shown as an example in Fig. S2. Thus, putative hybridization regions can be predicted for most of the identified target mRNAs. Furthermore, IntaRNA analysis with the N. meningitidis sibling sRNAs NmsR_A_ (NgncR_162) and NmsR_B_ (NgncR_163) revealed similar predictions for the orthologs of the *glyA* (NMB1055), *dadA* (NMB0176), NGFG_00249 (NMB1794), NGFG_00093 (NMB1964), NGFG_01937 (NMB2136), *norB* (NMB1622), *iscR* (NMB1378), *gloA* (NMB0340/NmsR_B_), and *gcvH* (NMB0575) putative targets.

10.1128/mbio.03093-22.1 FIG S1 *In silico* prediction of sRNA-mRNA interactions within the 5′ UTR. (a) Prediction of the secondary structure of NgncR_162 and NgncR_163 using the RNAfold web server (http://rna.tbi.univie.ac.at/cgi-bin/RNAWebSuite/RNAfold.cgi). Stem loops (SL) 1 to 3 and single-stranded regions (SSR) 1 and 2 are indicated. (b) Regions of complementarity between the sibling sRNAs and their target genes were analyzed with IntaRNA (27). In the case of *glyA*, NGFG_01937, and *norB*, predictions were identical for both siblings, while target-sRNA interactions shown for *dadA*, NGFG_00249, and NGFG_00093 apply to both NgncR_162 and NgncR_163 with only minor variations. Numbers refer to the nucleotide positions with respect to the translational start site (+1) in the case of mRNAs and the transcription initiation site in the case of NgncR_162 or NgncR_163. The start codon is marked in bold, and the RBS is underlined. Energy scores (E [kcal/mol]) from IntaRNA analysis are indicated. Download FIG S1, TIF file, 0.2 MB. Copyright © 2023 Steiner et al. 2023 Steiner et al. https://creativecommons.org/licenses/by/4.0/ This content is distributed under the terms of the Creative Commons Attribution 4.0 International license.

10.1128/mbio.03093-22.2 FIG S2 *In silico* prediction of sRNA-mRNA interactions within the coding region. Regions of complementarity between the sibling sRNAs and their target genes were analyzed with IntaRNA (27). In the case of NGFG_00045 and *iscR*, target-sRNA interactions apply to both NgncR_162 and NgncR_163 with only minor variations. In the case of *gloA*, complementarity to NgncR_163 is less pronounced and covers only nucleotides 209 to 226. Numbers refer to the nucleotide positions with respect to the translational start site (+1) in the case of mRNAs and the transcription initiation site in the case of NgncR_162 or NgncR_163. Energy scores (E [kcal/mol]) from IntaRNA analysis are indicated. Download FIG S2, TIF file, 0.2 MB. Copyright © 2023 Steiner et al. 2023 Steiner et al. https://creativecommons.org/licenses/by/4.0/ This content is distributed under the terms of the Creative Commons Attribution 4.0 International license.

### Validation of sRNA-target interactions on the protein level.

**FIG 3:**
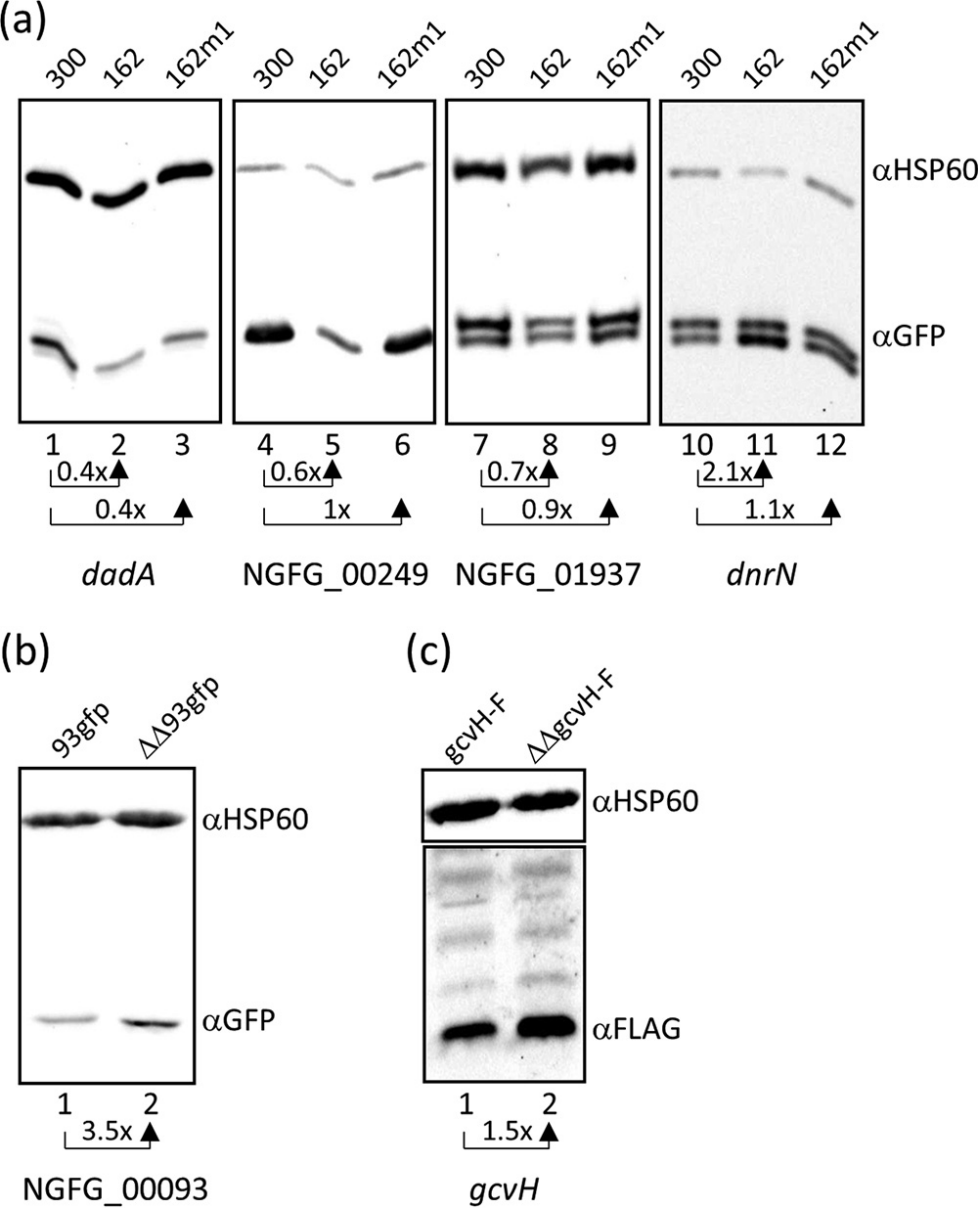
Validation of sibling sRNA/mRNA interactions on the protein level. (a) Expression of plasmid-borne translational target-*gfp* fusions (*dadA*-*gfp*, NGFG_00249-*gfp*, NGFG_01937-*gfp*, *dnrN*-*gfp*) was analyzed in E. coli Top10 cells, which were cotransformed with either plasmid pJV300 expressing a nonsense sRNA (lanes 1, 4, 7, and 10), NgncR_162 (pJV-162; lanes 2, 5, 8, and 11), or a derivative of NgncR_162 with mutated SL2 sequence (19) (pJV162m1; lanes 3, 6, 9, and 12). The figure shows the results of representative Western blot experiments (*n* = 2). For each target-*gfp* fusion, GFP levels (fold change) relative to E. coli cells harboring the negative-control plasmid pJV300 are indicated. (b) GFP expression was monitored by Western blotting in N. gonorrhoeae strains MS11 93*gfp* (lane 1) and MS11 ΔΔ93*gfp* (lane 2) harboring a translational NGFG_00093-*gfp* fusion inserted between the *iga* and *trpB* genes. The result from a representative experiment is shown (*n* = 2), and the relative change in GFP protein levels in the absence of the sibling sRNAs is indicated. (c) Expression of *gcvH* carrying a C-terminal FLAG tag was analyzed in the presence and absence of the sibling sRNAs in strains MS11 gcvH-F (lane 1) and MS11 ΔΔgcvH-F (lane 2). The result from a representative experiment is shown (*n* = 2), and the relative change in GFP expression is indicated. For Western blot analysis, equal amounts of protein were separated on 12% polyacrylamide gels and hybridization of membranes was performed with monoclonal antibodies directed against GFP (a and b) or FLAG (c). Hybridization with a monoclonal antibody directed against HSP60 was performed as a loading control.

Validation of sRNA-target interactions within the 5′ UTR was performed in Escherichia coli by using a two-plasmid *gfp* reporter system (28). The region covering the 5′ UTR and the first 8 to 32 codons of the NGFG_01937, NGFG_00249, and NGFG_00093, and *dnrN* genes was fused to *gfp*-SF encoding a superfolder variant of green fluorescent protein (GFP) in vector plasmid pXG10-SF (28). Similarly, the intergenic region between the NGFG_01721 and *dadA* genes as well as the first 40 codons of *dadA* was cloned into the intercistronic *gfp*-fusion vector pXG30-SF (28). E. coli Top10 cells were cotransformed with the target-*gfp* fusion plasmids, and plasmids expressing NgncR_162, a derivative of NgncR_162 with a mutated SL2 loop sequence (19), or a nonsense sRNA (29) and reporter gene expression was monitored by Western blotting. It should be noted that E. coli expressing NgncR_162 exhibited moderate growth retardation, as was also observed previously (19). While expression of NGFG_00093-*gfp* remained unaffected in E. coli (data not shown), downregulation of *gfp* expression was observed in the presence of NgncR_162 in the case of NGFG_01937 and NGFG_00249 and was abolished by mutagenesis of the SL2 loop sequence (Fig. 3a). GFP levels decreased to about 70% and 60%, respectively, of that observed in the negative control. Expression of *dadA*-*gfp* was diminished by 60% in the presence of NgncR_162, but in this case, the mutated SL2 loop sequence in sRNA NgncR_162m1 did not restore *dadA*-*gfp* expression to the level observed in the negative control (Fig. 3a). This observation is in accordance with the predicted sRNA-target interaction which, in the case of *dadA*, engages the SSR1 sequence (Fig. S1b). Interestingly, in accordance with transcriptome analysis, but despite the fact that sRNA binding is predicted to affect RBS accessibility (Fig. S1b), expression of *dnrN*-*gfp* was upregulated 2-fold in the presence of NgncR_162 (Fig. 3a). The mechanism of this unexpected regulation remains to be investigated further. Immunoblot results obtained from E. coli lysates could also be confirmed by direct fluorescence measurement (data not shown).

To investigate sRNA-mediated regulation of the protein level also in N. gonorrhoeae, NGFG_00093 and NGFG_00249 targets were chosen. Translational target-*gfp* fusions under the control of the respective target promoter were integrated into the intergenic region between the *iga* and *trpB* genes in wild-type MS11 and the ΔΔ162/163 mutant, and *gfp* expression in the mutants was monitored by Western blotting. Deep sequencing of the N. gonorrhoeae transcriptome had revealed only weak transcription of the NGFG_00249 gene (24), and consistently, NGFG_00249-GFP could not be detected (data not shown). Although NgncR_162 had no impact on the expression of NGFG_00093-*gfp* in E. coli, increased amounts of the fusion protein were detected in the absence of the sibling sRNAs in N. gonorrhoeae (Fig. 3b), confirming negative regulation via obstruction of the RBS. The observation that sRNA-mediated regulation failed in E. coli but was detectable in N. gonorrhoeae had previously also been made in the case of the sibling sRNA target *gdhR* (19). To validate predicted sRNA binding within the coding region, *gcvH* was selected (Fig. S2); this is the last gene in a tricistronic transcript composed of *gcvT* (NGFG_01512), the NGFG_01513 gene (encoding a hypothetical protein), and *gcvH* (24). Since transcript levels of *gcvT* and the NGFG_01513 gene remained unchanged in the sRNA ΔΔ162/163 double deletion mutant, we reasoned that sRNA-mediated indirect effects on transcription initiation are unlikely to account for the differential expression of *gcvH* in the absence of the sibling sRNAs. Therefore, derivatives of wild-type MS11 and the ΔΔ162/163 mutant expressing a C-terminally FLAG-tagged GcvH protein were constructed. In accordance with our transcriptome data (Table 1), GcvH was found to be upregulated 1.5-fold in the ΔΔgcvH-F mutant (Fig. 3c).

### The NGFG_00045 mRNA level is directly affected by the sibling sRNAs.

**FIG 4:**
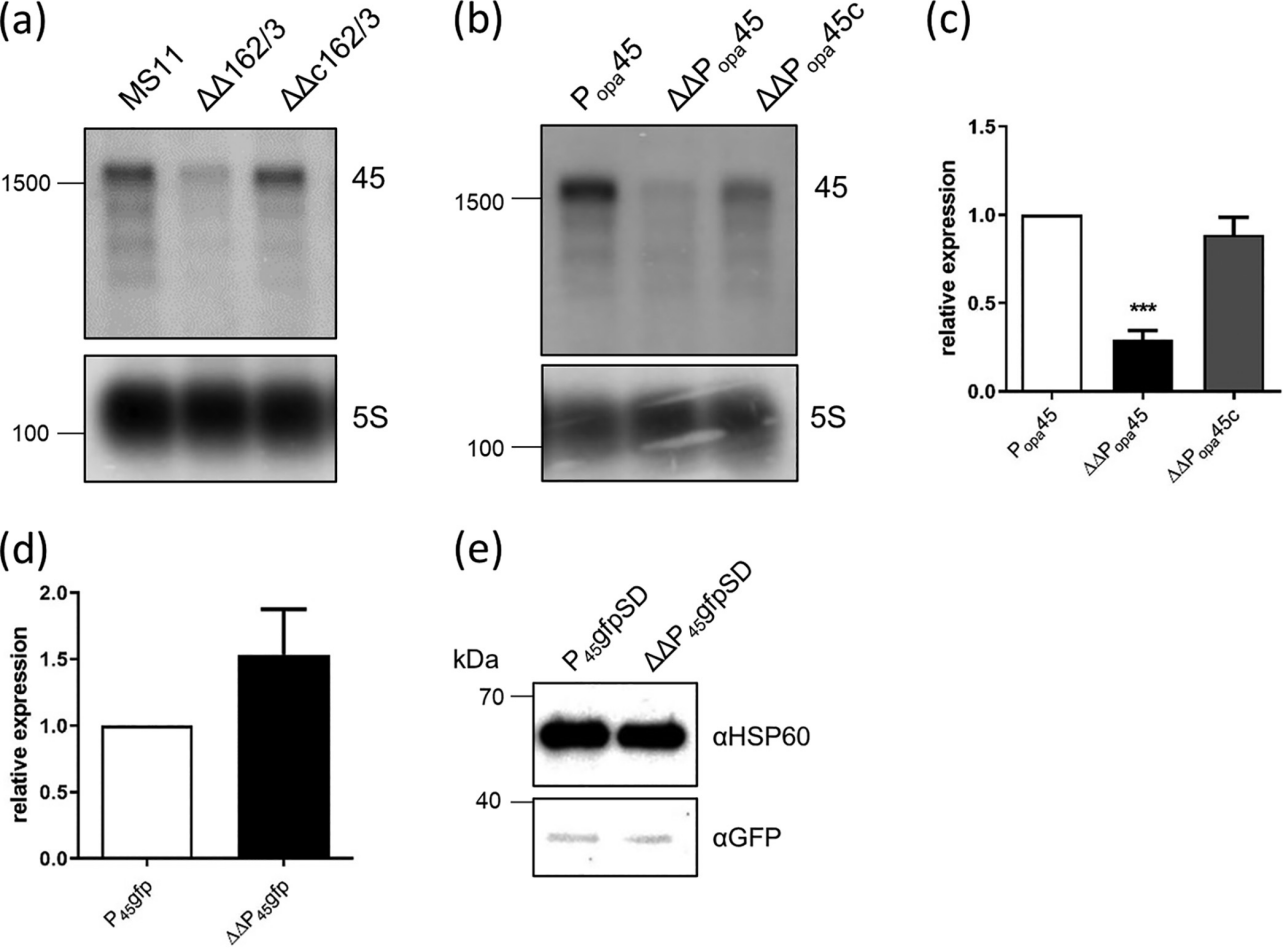
Expression analysis of NGFG_00045 in the presence and absence of the sibling sRNAs. (a and b) Equal amounts of RNA extracted from MS11, MS11 ΔΔ162/163, and MS11 ΔΔc162/163 (a) or MS11 P*_opa_*45, MS11 ΔΔP*_opa_*45, and MS11 ΔΔP*_opa_*45c (b) grown to logarithmic phase were analyzed by Northern blotting using a NGFG_00045-specific radiolabeled probe, which was generated by PCR using primer pair qRT45-1/qRT45-2 (see Table S2 in the supplemental material). Probing for 5S rRNA was used as a loading control. The positions of radiolabeled marker RNAs of 1,500 and 100 nt are indicated on the left side of the panels. (c) Transcription of NGFG_00045 under the control of the P*_opa_* promoter in MS11 P*_opa_*45, MS11 ΔΔP*_opa_*45, and MS11 ΔΔP*_opa_*45c was analyzed by qRT-PCR. The ratios of the transcript amount relative to that of P*_opa_*45 (normalized to 1) are depicted. The indicated ratios represent the mean of results of qRT-PCR experiments performed in triplicate on cDNAs obtained from three independent RNA preparations. Error bars indicate standard deviations. Statistical significance was determined using Student's *t* test analysis (***, *P* < 0.001). (d) Transcription of the reporter gene *gfp* in strains MS11 P_45_gfp and MS11 ΔΔP_45_gfp expressing *gfp* under the control of the NGFG_00045 promoter was quantified by qRT-PCR. The transcript amount in MS11 P_45_gfp was normalized to 1. The indicated ratio represents the mean of the results of qRT-PCR experiments performed in triplicate on cDNAs obtained from two independent RNA preparations. (e) Western blot analysis was performed on equal amounts of protein extracted from strains MS11 P_45_gfpSD and MS11 ΔΔP_45_gfpSD with monoclonal antibodies directed against GFP and HSP60 used as a loading control. Numbers on the left side of the panel indicate the positions of size marker proteins.

RNAs downregulated in the absence of NgncR_162/163 identified in this study represent a new class of targets. To demonstrate the validity of this class of targets, NSS family transporter NGFG_00045 was chosen to investigate this positive regulation by the sibling sRNAs in more detail. Northern blot analysis was performed on RNA extracted from MS11, the ΔΔ162/163 mutant, and the ΔΔc162/163 complemented mutant. RNA-seq had revealed that the NGFG_00045 gene is cotranscribed with a gene encoding a hypothetical peptide of 30 amino acids (24), and a transcript of the expected size was observed in wild-type MS11 and the complemented mutant but was barely detectable in the ΔΔ162/163 mutant (Fig. 4a). The hybridization pattern of RNA from the ΔΔ162/163 mutant was unaltered, indicating that the sibling sRNAs are not involved in prominent processing events (Fig. 4a). Next, we replaced the NGFG_00045 promoter region (24) with the promoter of a gonococcal *opa* gene in MS11 and the ΔΔ162/163 and ΔΔc162/163 mutants. qRT-PCR and Northern blot analysis showed that transcript amounts were still affected by the absence of the sibling sRNAs (Fig. 4b and c). However, when the NGFG_00045 upstream region including promoter and 5′ UTR was fused to *gfp*, almost equal amounts of mRNA were detected in the presence and absence of the sibling sRNAs (Fig. 4d). Since expression of GFP could not be detected in these mutants, probably due to a weak RBS in the NGFG_00045 5′ UTR, we introduced the consensus E. coli Shine-Dalgarno sequence via site-directed mutagenesis. As observed for *gfp* mRNA, protein levels were not affected in the absence of the sibling sRNAs (Fig. 4e). These data demonstrated that the positive regulation of NGFG_00045 is not indirectly mediated via a transcriptional regulator that is the target of the sRNAs and that the NGFG_00045 5′ UTR is not involved in posttranscriptional regulation. Rifampin assays performed on cultures of wild-type MS11, the ΔΔ162/163 mutant, and the ΔΔc162/163 complemented strain suggested a 30% reduction in NGFG_00045 transcript stability in the absence of the sibling sRNAs (Fig. S3). The mechanism of NGFG_00045 target activation by the sibling sRNAs remains to be investigated in more detail.

10.1128/mbio.03093-22.3 FIG S3 Determination of NGFG_00045 transcript stability. N. gonorrhoeae MS11, MS11 ΔΔ162/163, and MS11 ΔΔc162/163 were grown to an OD_550_ of 0.4 in PPM, and then rifampin was added to a final concentration of 100 μg/mL to block RNA synthesis (*t* = 0). At the indicated time points (*t* = 1, 3, 5, and 10 min), aliquots of the culture were removed, mixed with stop solution (95% [vol/vol] ethanol, 5% [vol/vol] phenol), and frozen in liquid nitrogen. RNA was prepared from the samples, and transcript amounts were determined by qRT-PCR. The transcript amount of the sample taken immediately after the addition of rifampin (*t* = 0) was normalized to 100%. The graphs depict the mean of data from two independent experiments. Calculated mRNA half-lives (*t*_1/2_) are 2.4 min in MS11 ΔΔ162/163 versus 3.6 min in MS11 ΔΔc162/163. Download FIG S3, TIF file, 0.2 MB. Copyright © 2023 Steiner et al. 2023 Steiner et al. https://creativecommons.org/licenses/by/4.0/ This content is distributed under the terms of the Creative Commons Attribution 4.0 International license.

10.1128/mbio.03093-22.10 TABLE S2 Oligonucleotides used in this study. ^a^Sequences introduced for cloning purposes are given in lowercase letters. Restriction sites are underlined. Download Table S2, DOCX file, 0.02 MB. Copyright © 2023 Steiner et al. 2023 Steiner et al. https://creativecommons.org/licenses/by/4.0/ This content is distributed under the terms of the Creative Commons Attribution 4.0 International license.

### Amino acid uptake is altered in the ΔΔ162/163 mutant and an NGFG_00045 deletion mutant.

**FIG 5:**
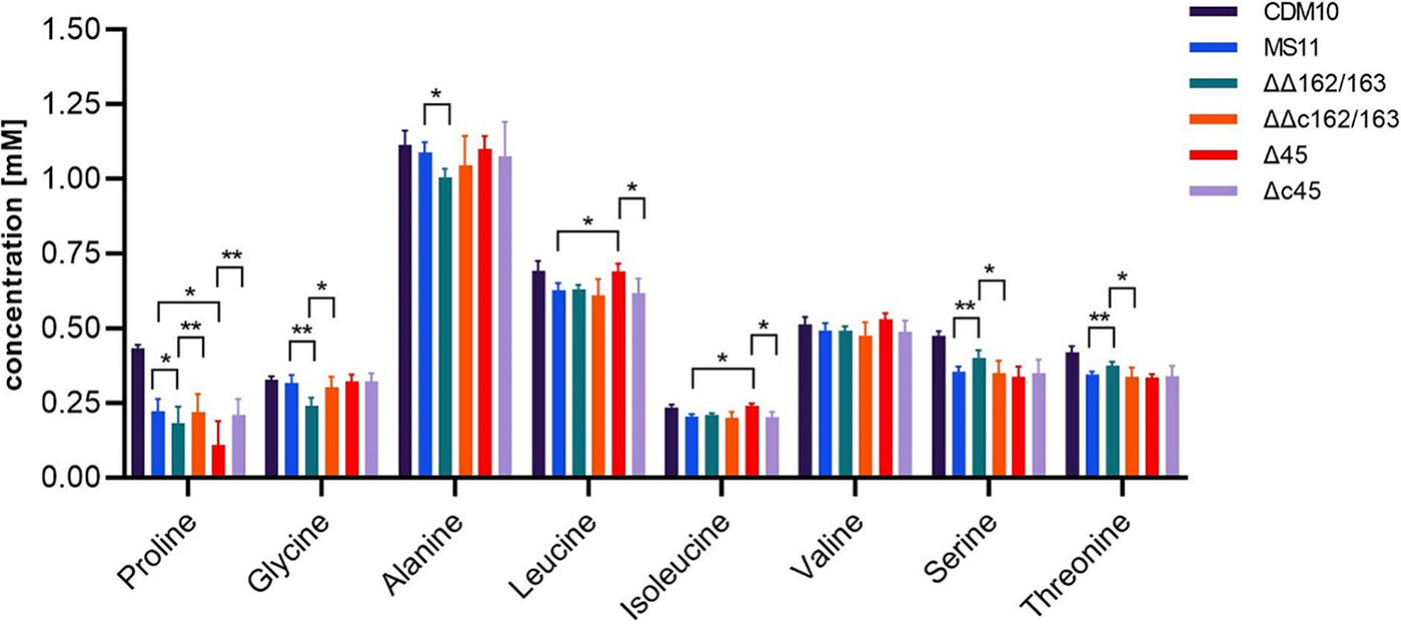
Analysis of the impact of sRNA and NGFG_00045 knockout on amino acid uptake by N. gonorrhoeae. N. gonorrhoeae MS11, MS11 ΔΔ162/163, MS11 ΔΔc162/163, MS11 Δ45, and MS11 Δc45 were grown to an OD_550_ of 0.6 in modified CDM10 medium. Bars depict the amino acid concentrations (mM) of culture supernatants of N. gonorrhoeae strains determined by mass spectrometry and represent the mean of four biological replicates (5 technical replicates each). For comparison, amino acid concentrations determined for CDM10 medium (see Fig. S6) are included. Error bars indicate standard deviations. Statistical significance was determined using Student's *t* test analysis (*, *P* < 0.05; **, *P* < 0.01).

Since several targets of the sibling sRNAs were predicted to be involved in transport processes, amino acid uptake was analyzed in the wild-type, the ΔΔ162/163 double deletion mutant, and the complemented strain to identify effects associated with sibling sRNA expression. First, the growth behavior of the respective N. gonorrhoeae strains was monitored in chemically defined CDM10 medium, demonstrating slightly reduced growth of the sRNA ΔΔ162/163 double deletion mutant (Fig. S4a). Furthermore, Northern blot analysis revealed abundant sRNA expression in gonococci grown in CDM10, with only a minor reduction compared to that of bacteria from PPM culture (Fig. S5). For the analysis of spent culture supernatants, gonococci were grown to logarithmic phase (optical density at 550 nm [OD_550_], 0.6) and amino acid levels were determined by ultraperformance liquid chromatography-mass spectrometry (UPLC/MS). Growth of wild-type MS11 resulted in 90% depletion of glutamine and glutamate and about 50% and 40% reduction of proline and asparagine concentrations, respectively, in the spent culture medium (Fig. S6). Consumption was marginal in the case of glycine, alanine, arginine, tyrosine, tryptophan, valine, and histidine (less than 5% of the supplied amounts) and moderate (ranging from 5% to 30%) for other amino acids. Surprisingly, the concentration of aspartate seemed to be even higher in the culture supernatant (Fig. S6), suggesting a lack of aspartate transport, which might be compensated by efficient biosynthesis of oxaloacetate from phosphoenolpyruvate (PEP) via PEP carboxylase (30); (see below) or deamination of asparagine. Compared to the wild-type, the sRNA ΔΔ162/163 double mutant showed significant differences in the consumption of proline, glycine, alanine, serine, and threonine. More specifically, consumption of glycine, alanine, and proline was increased 9-, 4-, and 1.2-fold, respectively, while uptake of serine and threonine was diminished about 1.5-fold. As expected, the amino acid profile of the culture supernatant of the ΔΔc162/163 complemented strain resembled that of the wild-type (Fig. 5).

10.1128/mbio.03093-22.4 FIG S4 Growth behavior of the sRNA ΔΔ162/163 double mutant and transporter knockout mutants in chemically defined CDM10 medium. N. gonorrhoeae wild-type strain MS11 and the respective mutants were grown at 37°C in PPM to an OD_500_ of 0.4. Gonococci were sedimented by centrifugation, bacteria were resuspended in 1 mL of CDM10 medium, and the optical densities of the inocula were determined. CDM10 medium was inoculated to an OD_550_ of 0.1, and the incubation was continued for 5 h. The optical density was monitored every hour. (a) Growth curves of the sRNA ΔΔ162/163 double mutant and the ΔΔc162/163 complemented strain are compared to that of wild-type MS11. (b) Growth of MS11 and the Δ45, Δ1564, and Δ1721 mutants which lack the NGFG_00045 and NGFG_01564 genes, encoding transporters of the neurotransmitter sodium symporter (NSS) family, and the NGFG_01721 gene, encoding a protein of the alanine or glycine:cation symporter (AGCS) family, respectively. Data points represent the mean of three independent experiments. Download FIG S4, TIF file, 0.4 MB. Copyright © 2023 Steiner et al. 2023 Steiner et al. https://creativecommons.org/licenses/by/4.0/ This content is distributed under the terms of the Creative Commons Attribution 4.0 International license.

10.1128/mbio.03093-22.5 FIG S5 Expression of the sibling sRNAs in rich PPM and chemically defined CDM10 medium. N. gonorrhoeae MS11 was precultured in PPM to an OD_550_ of 0.4. The preculture was divided, bacteria were sedimented by centrifugation, the pellets were resuspended in 1 mL of either PPM or CDM10 medium, and the optical densities of the inocula were determined. Main cultures in PPM and CDM10 medium were inoculated to a starting OD_550_ of 0.1, and bacteria were grown to mid-log phase. RNA was isolated and analyzed by Northern blotting using sRNA-specific radiolabeled oligonucleotide probes (NBS162 and NBS163) (Table S2). The result of a representative experiment is shown in panel a. Probing for 5S rRNA was used as a loading control. The positions of radiolabeled marker RNAs of 90 and 100 nt are indicated on the left side of the panels. In panel b, quantification of signal intensities of three independent experiments is shown. Sibling sRNA expression in rich PPM is normalized to 1. Download FIG S5, TIF file, 0.2 MB. Copyright © 2023 Steiner et al. 2023 Steiner et al. https://creativecommons.org/licenses/by/4.0/ This content is distributed under the terms of the Creative Commons Attribution 4.0 International license.

10.1128/mbio.03093-22.6 FIG S6 Amino acid consumption by N. gonorrhoeae MS11 grown in a chemically defined medium. N. gonorrhoeae MS11 was grown to an OD_550_ of 0.6 in modified CDM10 medium. Bars depict the amino acid concentration (mM) determined for CDM10 medium and spent medium from MS11 culture by mass spectrometry and represent the mean of four biological replicates (five technical replicates each). Error bars indicate the standard deviations. Statistical significance was determined using Student’s *t* test analysis (*, *P* < 0.05; **, *P* < 0.01; ***, *P* < 0.001). Download FIG S6, TIF file, 0.6 MB. Copyright © 2023 Steiner et al. 2023 Steiner et al. https://creativecommons.org/licenses/by/4.0/ This content is distributed under the terms of the Creative Commons Attribution 4.0 International license.

**FIG 6:**
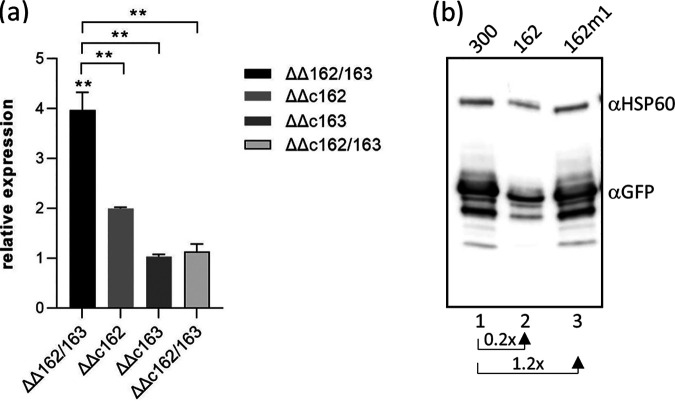
Posttranscriptional regulation of *glyA* by the sibling sRNAs NgncR_162/163. (a) GlyA transcript amounts were quantified by qRT-PCR in wild-type MS11, the ΔΔ162/163 mutant, the ΔΔc162 and ΔΔc163 single complementation mutants, and the ΔΔc162/163 double complemented strain. The ratios of the transcript amount relative to that of wild-type MS11 (normalized to 1) are depicted. Error bars indicate standard deviations (*n* = 3). Statistical significance was determined using Student's *t* test analysis (**, *P* < 0.01). (b) Expression of a translational *glyA*-*gfp* fusion was analyzed in E. coli in the absence and presence of sRNA NgncR_162. E. coli Top10 cells were cotransformed with plasmid pXG-863 (expressing a translational *glyA*-*gfp* fusion) and either plasmid pJV300 expressing a nonsense RNA (lane 1), or plasmids expressing NgncR_162 (pJV-162; lane 2), and a mutated derivative of NgncR_162 (pJV-162m1; lane 3). Equal amounts of protein were separated on a 12% polyacrylamide gel, and Western blot analysis was performed with monoclonal antibodies directed against GFP and HSP60 used as a loading control. The results shown are from a representative experiment (*n* = 2). GFP levels (fold change) relative to that of E. coli cells harboring the negative-control plasmid pJV300 are indicated.

To investigate the contribution of sibling sRNA targets to amino acid transport, knockout mutants for NGFG_01721, which belongs to the alanine or glycine:cation symporter (AGCS) family, and for the NSS family transporters NGFG_00045 and NGFG_01564 were created. Growth of the mutants was indistinguishable from that of the wild-type in rich medium (data not shown), but both the Δ45 and Δ1564 mutants exhibited a clear growth defect in CDM10 medium (Fig. S4b). The Δ1721 knockout mutant showed an intermediate phenotype resembling the growth of the sRNA double deletion mutant in CDM10 (Fig. S4b). In the NGFG_00045 Δ45 knockout mutant, proline uptake was massively increased, while the uptake of leucine, isoleucine, and valine was abolished. Again, the observed effects were reversed in the respective Δc45 complementation mutant (Fig. 5). Analysis of the amino acid compositions of culture supernatants of Δ1721 and Δ1564 mutants did not reveal statistically significant differences from that of wild-type MS11 (data not shown). In the Δ45 mutant, increased consumption of proline, which can be efficiently converted to glutamate via the bifunctional proline dehydrogenase/pyrroline-5-carboxylate dehydrogenase NGFG_01376, apparently compensates for the defect in isoleucine uptake caused by the inactivation of NGFG_00045, which is likely to be a BCAA transporter (for more details, see below). Elevated proline uptake in the ΔΔ162/163 mutant is consistent with downregulation of NGFG_00045 in this mutant. Furthermore, increased glycine uptake in the ΔΔ162/163 mutant strongly argues in favor of NGFG_01721 being predominantly a glycine transporter, since this protein is massively upregulated in the absence of the sibling sRNAs (Table 1) (19). Glycine cleavage, which is affected by the sibling sRNAs via target gene *gcvH*, yields 5,10-MTHF, which together with another molecule of glycine can then be converted to serine by the serine hydroxymethyltransferase GlyA (reviewed in reference 31) (see Fig. 10). In N. meningitidis, *glyA* expression was reported to be derepressed in the absence of *hfq* and the sibling sRNAs, respectively (21, 25). Since serine uptake was diminished in the ΔΔ162/163 mutant and in our RNA-seq analysis *glyA* was weakly upregulated with a *q* value close to significance (fold change, 1.29; *q* = 0.059), we validated *glyA* transcript levels by qRT-PCR. In fact, upregulation of *glyA* was observed in the sRNA double deletion mutant, while the wild-type transcript level was restored in the complemented strain (Fig. 6a). Furthermore, a translational *glyA*-*gfp* fusion was downregulated in the presence of NgncR_162 in E. coli (Fig. 6b), confirming negative regulation of *glyA* by the sibling sRNAs. This is in accordance with predicted binding of the sibling sRNAs to the RBS of the *glyA* mRNA (Fig. S1b). Based on these multiple evidences, we conclude that derepression of NGFG_01721, *gcvH*, and *glyA* in the sRNA double mutant enhances serine biosynthesis, resulting in a decreased demand for serine uptake.

### Isotopologue profiling reveals a bipartite metabolism in N. gonorrhoeae.

From all these findings, it became apparent that the sibling sRNAs in N. gonorrhoeae are involved in the regulation of the central carbon metabolism in response to available nutrients in the environment. The central carbon metabolism in N. gonorrhoeae has so far only been investigated using enzyme assays with cell extracts or genome annotations (32, 33). To generally define the central carbon metabolism of N. gonorrhoeae during growth and to further substantiate the effects of the sibling sRNAs, we here employed stable isotope incorporation experiments with subsequent isotopologue profiling.

To this aim, the bacteria were grown for 4 h in duplicate to logarithmic phase (OD_550_ = 0.6) in the chemically defined medium CDM10 containing fully ^13^C-labeled glucose or proline ([U-^13^C_6_]glucose or [U-^13^C_5_]proline). After harvest, the cells were mechanically disrupted (fraction 1) or hydrolyzed under acidic conditions (fraction 2). Protein-derived amino acids (in fraction 2) and fatty acids (in fraction 1) were silylated and then applied to gas chromatography (GC)-MS analysis (three technical replicates). From the relative masses detected for the specific fragments in the MS spectra, the ^13^C-excess values of amino acids and fatty acids were calculated using the software package Isotopo (34). Here, the overall ^13^C-excess values show ^13^C contents beyond the natural ^13^C abundances in the respective molecules. Moreover, using the same software, the isotopologue compositions were determined displaying the relative fractions (%) of isotopologues (M + 1, M + 2, M + 3, …, M + *n*) for each molecule under study. Herein, M denotes the molecular mass with only ^12^C in the carbon backbone of the molecule and *n* specifies the number of ^13^C-atoms.

**FIG 7:**
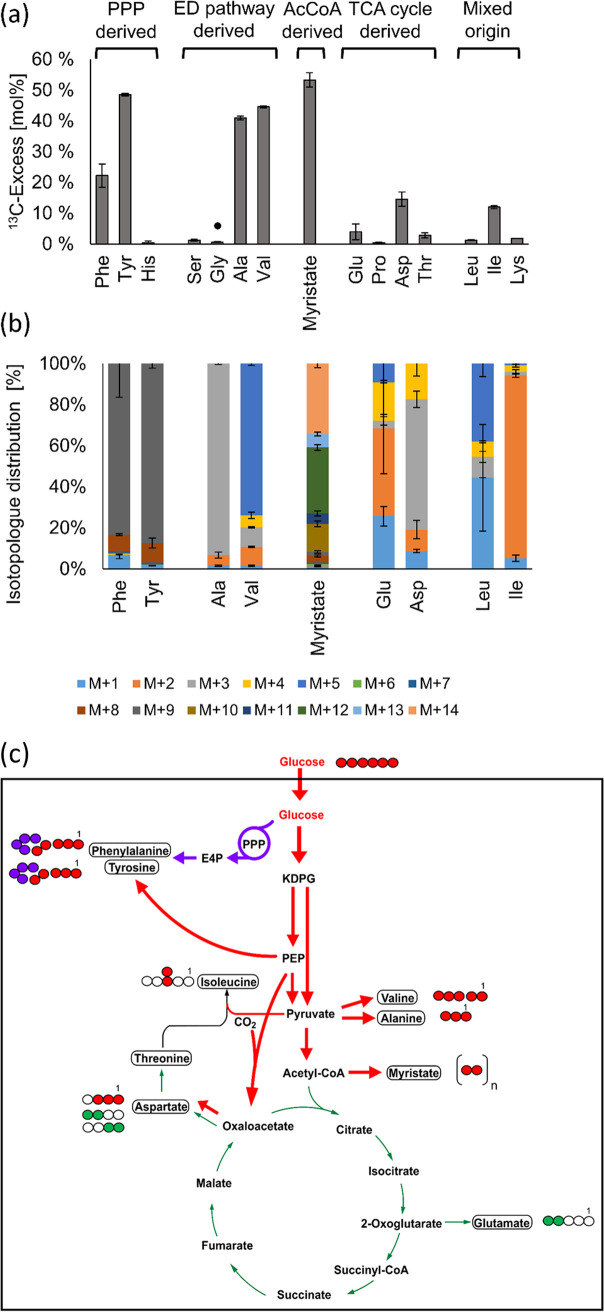
^13^C excess and isotopologue profiles of myristate and protein-derived amino acids from N. gonorrhoeae after growth in the presence of 13.9 mM [U-^13^C_6_]glucose. (a) The ^13^C excess (mol%) displays the overall ^13^C incorporation in the respective metabolite beyond natural ^13^C abundance. The amino acids are arranged in groups according to their relation to the central carbon metabolism. The values are means from two biological replicates and three technical replicates. The error bars indicate mean deviations. A solid black circle highlights glycine, which is not derived from a C_3_ intermediate but is rather formed from threonine (for details, see the text). (b) Isotopologue compositions of myristate and amino acids with significant ^13^C excess; M denotes the molecular mass with only ^12^C in the carbon backbone of the respective amino acid, while M + X represents the incorporation of the number (X) of ^13^C atoms. The values are means from two biological replicates and three technical replicates. The error bars indicate mean deviations. (c) Scheme of the carbon fluxes starting from the supplied [U-^13^C_6_]glucose in the central carbon metabolism of N. gonorrhoeae. The red arrows indicate fluxes into PEP and pyruvate via the ED pathway and into acetyl-CoA for fatty acid biosynthesis. Downstream reactions from these intermediates into amino acids and oxaloacetate are also indicated by red arrows. The purple arrows indicate fluxes via the PPP and the shikimate pathway, and the green arrows indicate fluxes from acetyl-CoA into the TCA and the downstream amino acids glutamate, aspartate, and threonine via citrate, 2-oxoglutarate, and oxaloacetate, respectively. The thickness of the arrows corresponds to the approximate relative extent of ^13^C incorporation from the labeled tracer. The boxes indicate metabolic products which were analyzed by GC-MS. The filled colored circles indicate ^13^C-labeled positions in these molecules, whereas the open circles indicate unlabeled positions.

Using 13.9 mM [U-^13^C_6_]glucose as a supplement to the medium, high ^13^C-excess values were detected in alanine (41.3% ^13^C excess) with about 90% M + 3 (i.e., displaying the fraction of the U-^13^C_3_-labeled species) and valine (44.7% ^13^C excess) with about 70% M + 5 (Fig. 7a and b). This confirmed the efficient uptake and utilization of [U-^13^C_6_]glucose to afford fully labeled pyruvate, which was then converted into the detected [U-^13^C_3_]alanine and [U-^13^C_5_]valine specimens, respectively (see also Fig. 7c). Although the labeling patterns from [U-^13^C_6_]glucose do not allow a distinction between glycolysis and the Enter-Doudoroff (ED) pathway for glucose degradation, the ED pathway is the predominant pathway for pyruvate formation from glucose in N. gonorrhoeae, based on enzyme assays (32). In addition, glycolysis seems to be nonfunctional due to the absence of the gene for phosphofructokinase in the genome of N. gonorrhoeae (33).

Tyrosine (^13^C excess of 48.6%) and phenylalanine (^13^C excess of 19.5%) also reflected the efficient incorporation of exogenous [^13^C]glucose via the ED pathway and the pentose phosphate pathway (PPP) producing M + 4 erythrose-4-phosphate (E-4-P) and M + 3 phosphoenolpyruvate (PEP) as precursors. More specifically, the formation of the detected M + 9 isotopologues in tyrosine and phenylalanine can be easily explained by the assembly of the aromatic amino acids from [U-^13^C_4_]E-4-P and two molecules of [U-^13^C_3_]PEP via the shikimate pathway (Fig. 7c). These observations are in full accordance with the reported genome sequence (33) and enzyme assays of sugar-degrading pathways (32).

Although serine is typically derived from 3-phosphoglycerate via 3-hydroxypyruvate, the genome of N. gonorrhoeae does not encode an enzyme for the 3-phosphoglycerate reduction into 3-hydroxypyruvate. Indeed, only a minor ^13^C excess (1.4%) was observed for serine from the protein hydrolysate in the [U-^13^C_6_]glucose experiment (Fig. 7a). In conclusion, serine was derived mainly from unlabeled serine and other unlabeled components in the medium. This was also in accordance with high serine uptake observed through culture supernatant analysis (Fig. S6). Not surprisingly then, almost no ^13^C excess was detected in glycine (0.7%), since glycine was derived from serine or was also directly taken up from the medium in an unlabeled form. Similarly, due to the absence of the histidinol phosphatase gene in the genome sequence, no ^13^C excess was detected in histidine.

The high ^13^C excess of the fatty acid myristate (about 55%) reflected the conversion of M + 3 pyruvate into M + 2 acetyl coenzyme A (acetyl-CoA), a precursor of fatty acid biosynthesis. Incorporation of M + 2 acetyl-CoA as a building block was detected as a mixture of ^13^C-isotopologues in myristate mainly with even numbers of ^13^C atoms (i.e., M + 8, M + 10, M + 12, and M + 14), indicating fatty acid biosynthesis using 4, 5, 6, or 7 [U-^13^C_2_]acetyl-CoA units, respectively (Fig. 7b). A smaller amount of M + 2-labeled acetyl-CoA was channeled into the oxidative TCA cycle (33, 35) via synthesis of citrate, which was then metabolized to [4,5-^13^C_2_]2-oxoglutarate and [4,5-^13^C_2_]glutamate (3.9%) (Fig. 7). In contrast, a higher ^13^C enrichment occurred in aspartate (14.6%), mainly due to the high relative fractions of M + 3 (about 60%) and M + 4 (about 20%) (Fig. 7). Based on these results, it can be assumed that PEP carboxylase (30) was highly active in converting [U-^13^C_3_]PEP and ^12^CO_2_ or ^13^CO_2_ into [1,2,3-^13^C_3_]oxaloacetate or [U-^13^C_4_]oxaloacetate, which were further converted to the observed [1,2,3-^13^C_3_]- and [U-^13^C_4_]aspartate species, respectively (Fig. 7b). ^13^CO_2_ was potentially produced directly in the cell during decarboxylation of [U-^13^C_3_]pyruvate to [1,2-^13^C_2_]acetyl-CoA and could therefore have locally produced a ^13^C enrichment in CO_2_ beyond natural abundance. Notably, only small amounts of aspartate seemed to be synthetized via the TCA cycle, since TCA-derived [^13^C_2_]aspartate accounted for only 10% of its isotopologue profile (Fig. 7b).

Additionally, some ^13^C label was observed in isoleucine (12%) by incorporation of a labeled pyruvate unit into C-3 and the attached methyl group (Fig. 7). No significant ^13^C excess was detected in leucine (1.1%), threonine (2.2%), lysine (1.8%), or proline (0.5%), although enzymes for the biosynthesis of proline from glutamate were annotated in the genome (33).

**FIG 8:**
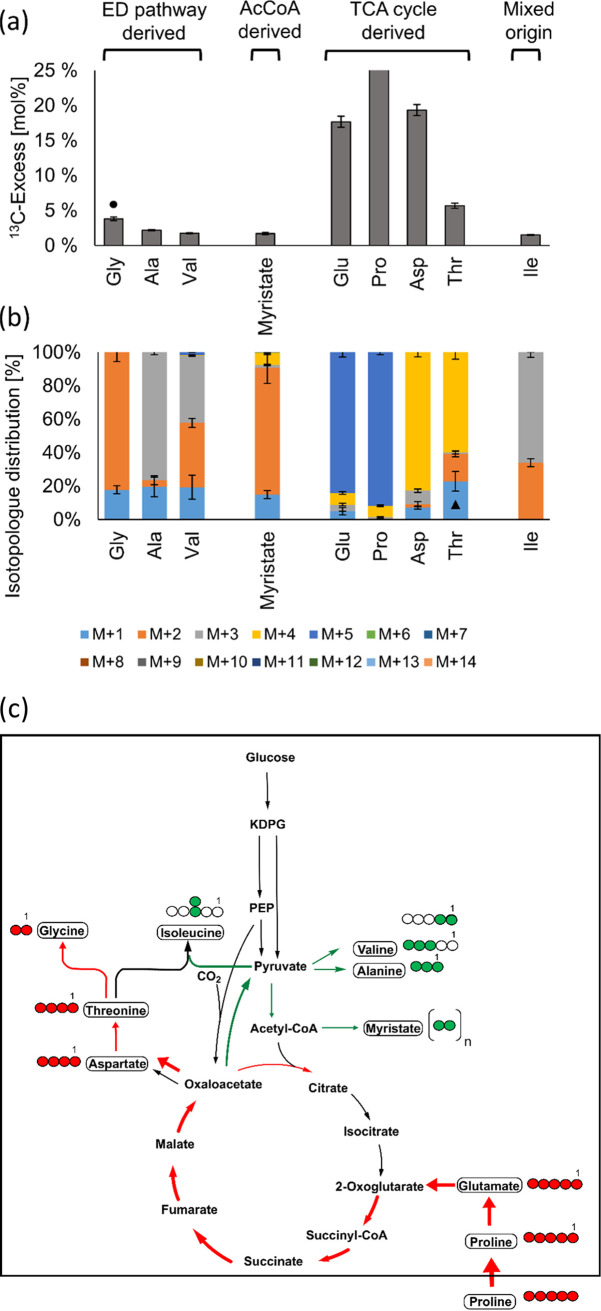
^13^C excess and isotopologue profiles of myristate and protein-derived amino acids from N. gonorrhoeae after growth in the presence of 0.4 mM [U-^13^C_5_]proline. (a) The ^13^C excess (mol%) displays the overall ^13^C incorporation in the respective metabolite beyond natural ^13^C abundance. (b) Isotopologue compositions of myristate and amino acids with significant ^13^C excess. A black triangle highlights M + 1 from threonine, which might be overestimated due to the presence of another substance with the same retention time from the GC column and having the same mass as M + 1 of threonine. (c) Scheme of the carbon fluxes starting from the supplied [U-^13^C_5_]proline in the central carbon metabolism of N. gonorrhoeae. The red arrows indicate fluxes via conversion of proline into glutamate, which feeds into 2-oxoglutarate and downstream intermediates of the TCA cycle. The green arrows indicate fluxes from oxaloacetate into pyruvate and amino acids thereof. Notably, no significant ^13^C excess was detected for metabolites further upstream from pyruvate. For more details, see the legend for Fig. 7.

Since only low ^13^C enrichments were detected in amino acids using intermediates of the TCA cycle in these experiments, we assumed that the TCA in N. gonorrhoeae is mainly fueled by unlabeled compounds from the medium (i.e., not from [U-^13^C_6_]glucose). The ΔΔ162/163 mutant had revealed an increased uptake of proline (Fig. 5), which indicated that this amino acid could serve as a precursor for glutamate and TCA cycle intermediates via 2-oxoglutarate. Therefore, a labeling experiment starting with 0.4 mM [U-^13^C_5_]proline in the medium was performed. Indeed, [U-^13^C_5_]proline added to the culture medium was efficiently taken up and utilized, leading to 80% M + 5 in the isotopologue profile of glutamate (17.7% ^13^C excess) and about 80% M + 4 in the isotopologue profiles of aspartate (20% ^13^C excess) and threonine (5.7% ^13^C excess) (Fig. 8). In contrast, only a low ^13^C excess was detected in alanine, glycine, valine, and isoleucine (below 5%), whereas serine did not contain ^13^C beyond the natural abundance content. Based on this observation, labeled glycine could hardly be formed from the apparently unlabeled serine. Rather, glycine was potentially synthesized from (labeled) threonine via the threonine utilization (Tut) pathway (reviewed in reference 31), as the genome of N. gonorrhoeae contains distant homologues of threonine dehydrogenase and 2-amino-3-ketobutyrate CoA ligase from E. coli.

**FIG 9:**
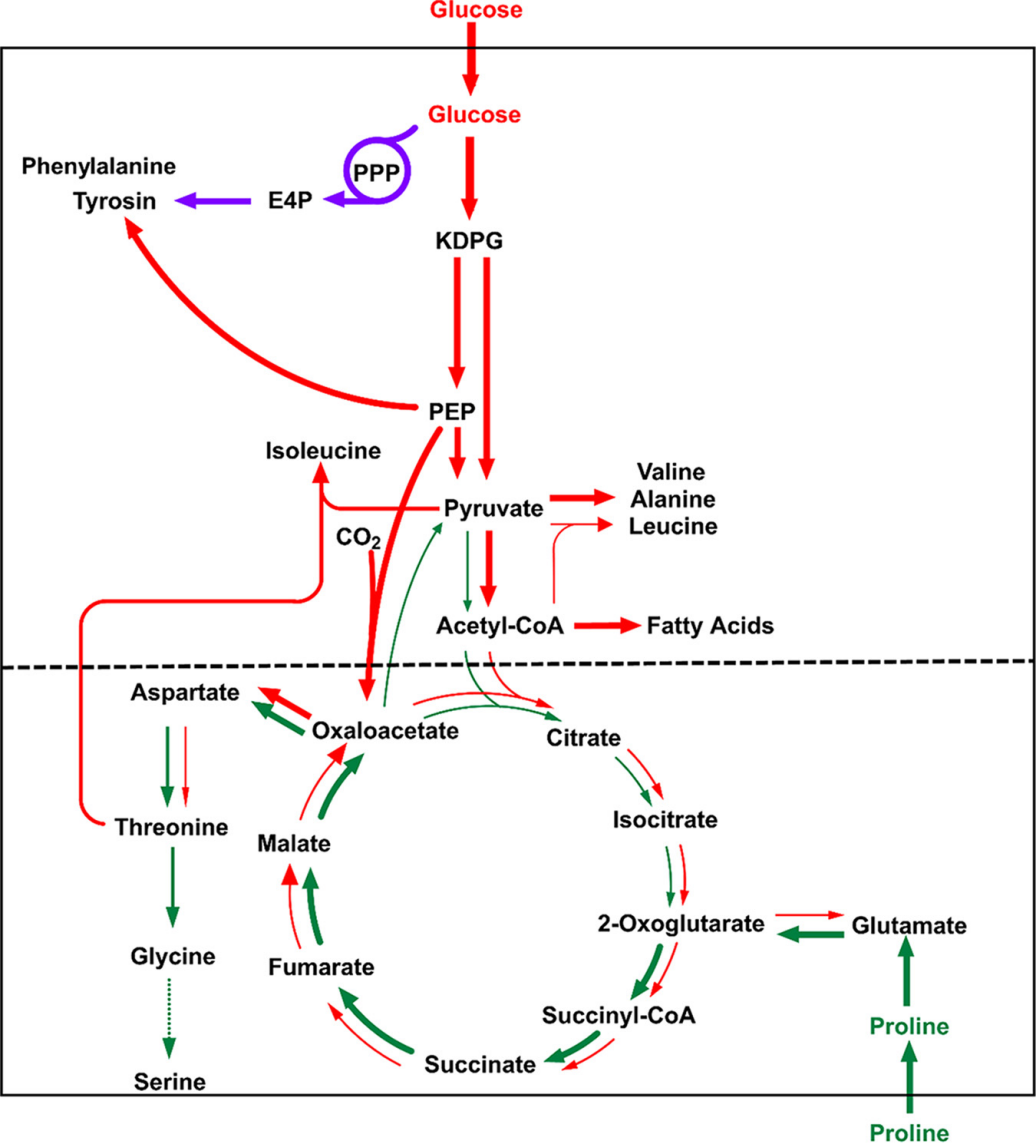
Model for the bipartite central carbon metabolism in N. gonorrhoeae. Red arrows indicate fluxes based on glucose utilization, purple arrows indicate fluxes via the PPP and the shikimate pathway, and green arrows indicate fluxes determined by the usage of proline and related amino acids. The thickness of the arrows corresponds to the approximate relative extent of ^13^C incorporation that was observed with the respective labeled tracers. The dashed black line indicates the borderline of the two metabolic modules in the bipartite metabolic network.

The different ^13^C enrichments from the labeling experiments with [^13^C]glucose and [^13^C]proline indicated that N. gonorrhoeae used different substrates simultaneously for its growth, suggesting a bipartite metabolic network, as shown in Fig. 9. In this model, glucose is used as a substrate providing precursors for anabolic purposes, i.e., for sugar components and some aromatic amino acids derived from the PPP, amino acids derived from pyruvate, and fatty acids derived from acetyl-CoA. In contrast, glucose does not serve as a major substrate to feed the TCA cycle. Rather, proline or related compounds drive the TCA cycle and contribute mainly to energy metabolism.

Based on this metabolic model, we then characterized the impact of the sibling sRNAs and targeted transport proteins on the core metabolism of N. gonorrhoeae. This was done by supplementing the respective mutant strains with [U-^13^C_6_]glucose or [U-^13^C_5_]proline during growth, as explained above for the parent strain.

### NGFG_00045 and NGFG_01564 transport BCAA and phenylalanine, respectively.

**FIG 10:**
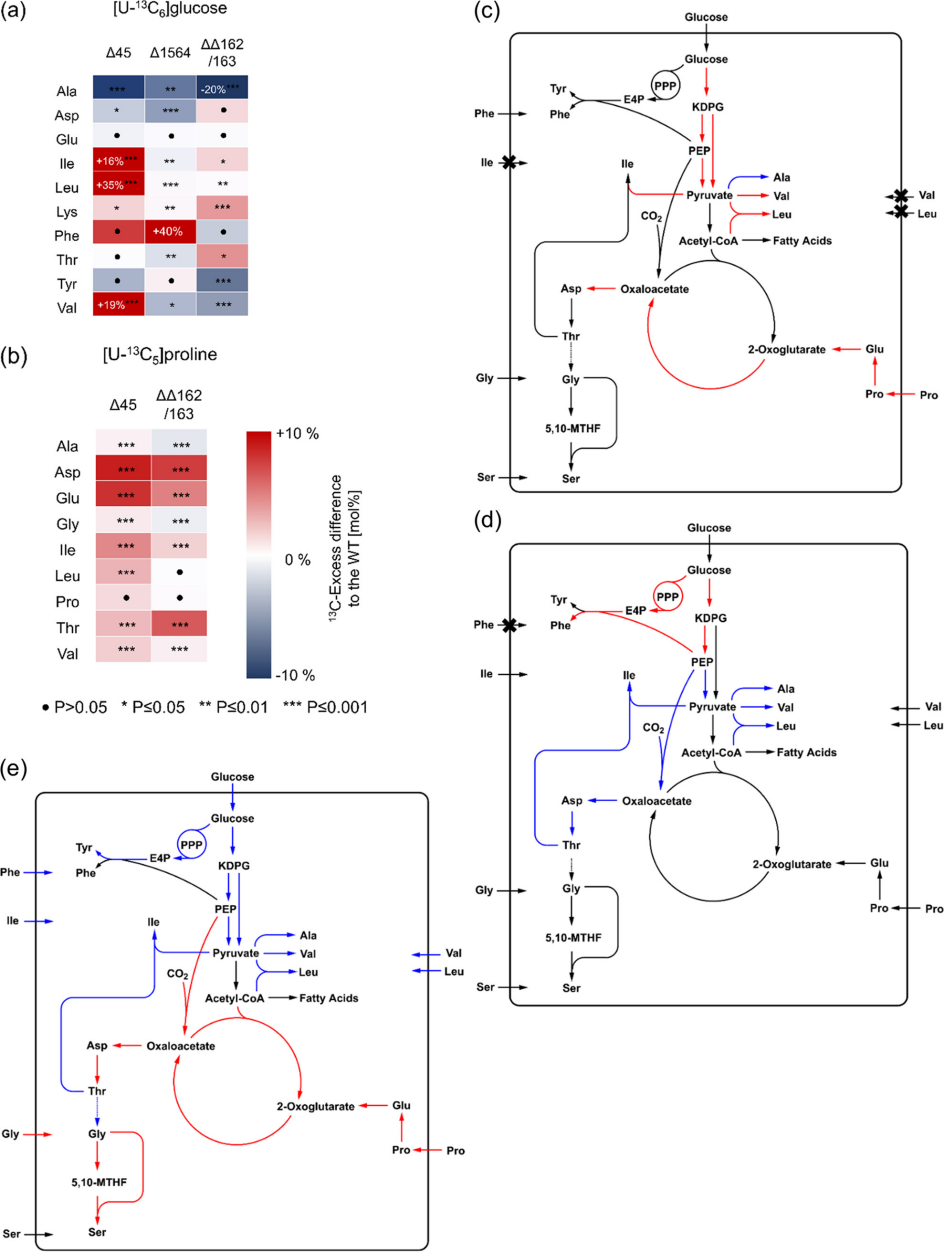
Differential labeling patterns and fluxes into protein-derived amino acids of N. gonorrhoeae MS11 (wild-type) and several mutant strains. (a) Heat map of ^13^C excess from [U-^13^C_6_]glucose in amino acids from N. gonorrhoeae ΔΔ162/163, Δ45, and Δ1564 mutants relative to the wild-type. (b) Heat map of ^13^C excess from [U-^13^C_5_]proline in amino acids from N. gonorrhoeae ΔΔ162/163 and Δ45 mutants relative to the wild-type. The numbers in the boxes indicate values outside the color scale. Statistical significance was determined using Student's *t* test analysis (filled circles, not significant; *, *P* < 0.05; **, *P* < 0.01; ***, *P* < 0.001). (c) Differential fluxes in the N. gonorrhoeae Δ45 mutant relative to the wild-type strain. Red arrows indicate upregulated fluxes, while blue arrows indicate downregulated fluxes. The crosses indicate downregulated uptake of amino acids in the mutant. (d) Differential fluxes in the N. gonorrhoeae Δ1564 mutant relative to the wild-type strain. Red arrows indicate upregulated fluxes, while blue arrows indicate downregulated fluxes. The crosses indicate downregulated uptake of phenylalanine in the mutant. (e) Differential fluxes in the N. gonorrhoeae ΔΔ162/163 double mutant relative to the wild type. Red arrows indicate upregulated fluxes, while blue arrows indicate downregulated fluxes and uptake of amino acids.

The sibling sRNA NGFG_00045 and NGFG_01564 target genes presumably encode amino acid transporters (Table 1; Fig. 5). Indeed, the ^13^C excesses of alanine, isoleucine, leucine, and valine differed significantly between the N. gonorrhoeae Δ45 mutant and the wild-type during growth with [U-^13^C_6_]glucose (Fig. 10a). While the ^13^C excess increased massively in the BCAA isoleucine, leucine, and valine (+16% to +35%), it decreased in alanine (−9%). The same effect, albeit to a much lower extent, was observed during growth of N. gonorrhoeae Δ45 with [U-^13^C_5_]proline (Fig. 10b). Here, other amino acids also showed minor differences, but the most significant effect was observed in glutamate and aspartate, with increased ^13^C excess values of 8 to 9% in the mutant. These complementary effects observed with different labeled substrates again corroborated the validity of the bipartite metabolic model introduced above (Fig. 9). Based on these observations, the NGFG_00045 gene encodes a BCAA transporter, as *de novo* synthesis of this class of amino acids, especially from [U-^13^C_6_]glucose, was highly increased in the Δ45 mutant strain. BCAAs use pyruvate as a building block (cf. Fig. 9); their increased biosynthesis depleted the pool of pyruvate available for alanine synthesis and thereby reduced the ^13^C excess in this amino acid. On the other hand, the synthesis of aspartate and glutamate was potentially upregulated to meet the increasing demand for aspartate as a precursor in isoleucine biosynthesis (cf. Fig. 9) and for nitrogen donors in the transamination step of BCAA biosynthesis (Fig. 10c).

In the case of the NGFG_01564 mutant (Δ1564), the labeling experiment with [U-^13^C_6_]glucose produced a huge increase in the ^13^C excess of phenylalanine (+40%) compared to that of the wild-type (Fig. 10a). Other significant differences (>1%) included a decrease in the ^13^C excess of alanine, valine, aspartate, and threonine (−6.2 to −1.6%). These effects could again be explained by a decreased pool of labeled PEP and pyruvate, as [^13^C]PEP was consumed for *de novo* phenylalanine biosynthesis in the Δ1564 mutant strain. As explained earlier, synthesis of aspartate from glucose heavily depends on the PEP carboxylase reaction in N. gonorrhoeae and aspartate is a precursor for threonine biosynthesis (cf. Fig. 9). Surprisingly, the ^13^C content of tyrosine was not significantly altered in the Δ1564 mutant strain (Fig. 10a), despite its origination from the same metabolic precursors as phenylalanine. Based on these observations, the NGFG_01564 gene encodes a phenylalanine transporter (Fig. 10d).

The identification of a previously noncharacterized BCAA and phenylalanine transporter as targets of the sibling sRNAs demonstrates the straightforward approach of our strategy to include ^13^C-based metabolomics as a tool to functionally analyze bacterial regulatory RNAs.

### Sibling sRNAs interfere with the TCA cycle, amino acid transport, and metabolism.

Looking at the sibling sRNA double mutant strain N. gonorrhoeae ΔΔ162/163, a variety of effects were observed in the labeling experiments. When supplementation was with [U-^13^C_6_]glucose (Fig. 10a), the ^13^C excess in alanine, tyrosine, and valine decreased (−5 to −20%), while the ^13^C excess in isoleucine, lysine, and threonine increased (+1.7 to +4%). In the experiment with [U-^13^C_5_]proline, the double mutant strain showed an increased ^13^C excess in aspartate, glutamate, and again in threonine and isoleucine (+5 to +7.6%). Alanine and glycine showed decreases in ^13^C excess of 1.1% and 0.7%, respectively (Fig. 10b).

As suggested earlier, the sibling sRNAs downregulate the TCA cycle activity via negative control of various enzymes in the cycle (19, 21). Consequently, in the double mutant strain, the TCA cycle was more active and utilized the supplied [^13^C]proline tracer at higher rates, thereby producing higher ^13^C excess values in aspartate and glutamate, which are derived from oxaloacetate and proline, respectively (cf. Fig. 9). By closer examination of the isotopologue profiles, this effect can be distinguished from the concomitant increased proline metabolization as a consequence of decreased BCAA uptake due to downregulation of NGFG_00045 (Fig. S7).

10.1128/mbio.03093-22.7 FIG S7 Isotopologue profiles of glutamate (a) and aspartate (b) from wild-type MS11, MS11 ΔΔ162/163, and MS11 Δ45 after growth in CDM10 medium supplemented with 13.9 mM [U-^13^C_6_]glucose. Analysis of the isotopologue profiles of aspartate and glutamate allows for the differentiation of metabolic effects in the ΔΔ162/163 and Δ45 mutants. In the Δ45 mutant, proline uptake is increased but expression of several TCA cycle enzymes is still repressed by the sibling sRNAs. When [U-^13^C_6_]glucose was supplemented as a tracer, the abundance of heavier isotopologues (>M + 2) in aspartate and glutamate decreased, as the labeling in the TCA cycle was diluted due to the increased uptake of unlabeled proline in comparison to that of the wild type. This lowered the probability for the combination of two labeled precursors that would potentially produce heavier isotopologues in aspartate and glutamate. In the ΔΔ162/163 mutant strain, a similar effect albeit to a lower extent is expected, as the expression of NGFG_00045 is no longer upregulated by the sibling sRNAs. However, as the TCA cycle is also no longer repressed, more labeled precursors entered the cycle and subsequently produced isotopologue profiles in aspartate and glutamate that were similar to that of the wild type. Download FIG S7, TIF file, 0.1 MB. Copyright © 2023 Steiner et al. 2023 Steiner et al. https://creativecommons.org/licenses/by/4.0/ This content is distributed under the terms of the Creative Commons Attribution 4.0 International license.

In the experiment with [^13^C]glucose, utilization of this substrate via the ED pathway as well as the PPP seems to be decreased, leading to lower ^13^C excess values in tyrosine and alanine. However, there was no significant effect observed in phenylalanine. Since the sibling sRNAs upregulate the phenylalanine transporter NGFG_01564, there was lower import of this amino acid in the double mutant strain. Therefore, the ^13^C excess was much less diluted by the import of exogenous phenylalanine than with the wild-type. The same case can be made for the BCAAs valine, leucine, and isoleucine, which were much less affected than alanine in the double mutant strain, although they are also fully or partially derived from pyruvate. Herein, the sibling sRNAs upregulated the BCAA transporter NGFG_00045, and consequently, reduced import was observed in the double mutant strain. However, genes in BCAA biosynthesis were also downregulated in the ΔΔ162/163 mutant (Table 1), which could explain the lower ^13^C excess in valine from [^13^C]glucose. Reduced glucose usage could be due to reduced glucose uptake, since mRNA levels of the putative glucose transporter NGFG_02263 were reduced in the sibling sRNA mutant (Table 1). Furthermore, increased uptake of unlabeled alanine by the sRNA double mutant due to the upregulation of NGFG_01721 (Fig. 5) is likely to contribute to reduced ^13^C accumulation in alanine.

Threonine presents an interesting case; when [^13^C]proline was supplemented, the ^13^C excess of threonine increased but to a smaller extent than that of its metabolic precursor aspartate. However, when [^13^C]glucose was used as a substrate, threonine showed a greater increase in ^13^C excess than aspartate. Increased ^13^C excess might be related to threonine acting as a precursor for glycine, which can be converted to serine by GlyA (reviewed in reference 31). Increased uptake of unlabeled glycine in the ΔΔ162/163 mutant (due to the upregulation of NGFG_01721) and elevated *glyA* expression resulted in an increased rate of serine production. These effects led to decreased ^13^C excess in glycine and also mitigated the need for threonine as a precursor for glycine/serine, thereby leading to the accumulation of labeled threonine in the double mutant strain. This is in accordance with the labeling experiments, since in the double mutant strain, the ^13^C excess in threonine was significantly increased while the ^13^C excess in glycine was significantly decreased with supplementation of [U-^13^C_5_]proline (Fig. 10b). The metabolic effects of sibling sRNA deletion are summarized in Fig. 10e.

## DISCUSSION

The functional analysis of regulatory RNAs is hampered by the fact that large regulons are often governed by them. Target identification by *in silico* predictions, quantification of mRNA or protein abundance in the presence or absence of the sRNA, or RNA-seq-based approaches exploiting direct sRNA-target mRNA interactions frequently yield numerous candidates, which are then used to predict the function of the sRNA. Since sRNA-mediated modifications of mRNA or protein abundances are often moderate and metabolic pathways are frequently controlled by posttranslational modifications rather than protein abundance, very little is known about the true regulatory impact of the rapidly growing number of sRNAs with predicted metabolic targets (36–41). We here performed a combined RNA-seq and metabolomics approach and thereby significantly extended the regulon controlled by the N. gonorrhoeae sibling sRNAs NgncR_162/163.

Our metabolomics analyses focused on differentially transported metabolites (amino acids), leading to identification of functions of heretofore uncharacterized NgncR_162/163 targets like the potential BCAA and phenylalanine transporters (NGFG_00045 and NGFG_01564, respectively). Metabolic flux analysis using isotopologue profiling confirmed the role of identified targets in metabolic pathways like the TCA and unveiled their role in orchestrating the substrates used in a newly identified bipartite metabolic network. The combination of transcriptome and carbon flux analyses used here thus provides an excellent approach toward a real understanding of the outcome of riboregulation targeting metabolic processes in bacteria.

New members of the NgncR_162/163 regulon predominantly encode proteins involved in nutrient uptake and serine/glycine metabolism. This is reminiscent of E. coli/Salmonella enterica serovar Typhimurium GcvB, which is conserved in members of the *Gammaproteobacteria* (36) and directly controls more than 50 mRNA targets comprising mostly periplasmic amino acid binding ABC transporter components, amino acid permeases (among others, the glycine permease encoded by *cycA*), and enzymes involved in amino acid metabolism (42–44). GcvB is abundant during exponential growth in nutrient-rich medium but hardly detectable in bacteria from stationary phase or upon culture in minimal medium (36, 45, 46). The growth phase-dependent expression pattern of GcvB results from the accumulation of the GcvB sponge SroC in stationary phase, which triggers the RNase E-dependent degradation of GcvB via a base-pairing interaction (43, 47). In addition, expression of GcvB is under the control of GcvA, the transcriptional regulator of the glycine cleavage operon *gcvTHP* (48), and is induced when glycine is available. As with GcvB, the *Neisseria* sibling sRNAs are most abundant during exponential growth under nutrient-rich conditions, while sRNA levels decline in stationary phase (see Fig. S8 in the supplemental material). Interestingly, in *Rhizobiales*, a plethora of ABC transport systems is regulated by sibling sRNAs (AbcR1 and AbcR2) (reviewed in reference 37).

10.1128/mbio.03093-22.8 FIG S8 The sibling sRNAs NgncR_162/163 are less abundant in stationary growth phase. (a) Northern blot analysis was performed on RNA extracted from bacteria from logarithmic phase, the transition phase between logarithmic growth and stationary phase, and stationary phase by using radioactively labeled sRNA-specific oligonucleotides. Probing for 5S rRNA was used as a loading control. The position of a radiolabeled marker RNA of 100 nt is indicated on the left side of the panel. Images from four independent experiments were used for quantification of sRNA amounts. (b) The growth curve of N. gonorrhoeae MS11 is shown, and time points at which samples for RNA preparation were taken are indicated. (c) The mRNA abundance of the NGFG_01721 target, which is negatively regulated by the sibling sRNAs, was quantified by qRT-PCR in bacteria grown to logarithmic and stationary phases, respectively. Transcript amounts present in bacteria from logarithmic phase were normalized to 1. qRT-PCR analysis was performed on cDNA from two independent RNA preparations. Download FIG S8, TIF file, 0.5 MB. Copyright © 2023 Steiner et al. 2023 Steiner et al. https://creativecommons.org/licenses/by/4.0/ This content is distributed under the terms of the Creative Commons Attribution 4.0 International license.

Newly identified targets are under both negative and positive control, indicating that the sibling sRNAs not only act by obstructing the RBS as demonstrated previously (19–21) but also employ other means of target regulation triggered by binding of the mRNA within the CDS (Fig. 4; Fig. S2). *In silico*-predicted regions of complementarity between the newly identified target mRNAs and the N. gonorrhoeae sibling sRNAs engage sequence motifs which are shared by both sRNA molecules (Fig. S1 and S2). However, in contrast to the well-established *prpB*, *prpC*, *ack*, and NGFG_01721 targets, full posttranscriptional regulation of which requires only one sibling (19; J. Helmreich and D. Beier, unpublished), complete functional redundancy is not observed for new members of the NgncR_162/163 regulon. Single complementation of MS11 ΔΔ162/163 under steady-state conditions revealed a higher impact of NgncR_163 on target regulation (Fig. 2b), which might be explained by the higher abundance of NgncR_163 (19). Such a diverse regulatory impact of siblings that differ in their abundances but exhibit the same base-pairing capability for the target has also been observed in the case of posttranscriptional regulation of the salmochelin siderophore receptor IroN by Salmonella Typhimurium RhyB1 and RhyB2 (49). Our findings therefore suggest hierarchical target control based on the abundance of the *Neisseria* sibling sRNAs. However, besides growth phase-dependent differences in sRNA abundancy, which similarly apply to both siblings (Fig. S8), environmental conditions affecting expression of NgncR_162/163 could not yet be identified.

From isotopologue profiling experiments, we deduced a bipartite metabolism in N. gonorrhoeae, where energy metabolism is driven mainly via amino acids like glutamate and proline, which feed the TCA cycle, while glucose degradation via the ED and PPP pathway mostly provides intermediates for anabolic pathways (Fig. 9). This model is well in line with the observation that glutamate, glutamine, and proline (and also asparagine) are consumed to the highest extent when gonococci are grown in chemically defined media (Fig. S6). Similar models for a bipartite metabolic network have already been presented for other pathogenic bacteria such as Listeria monocytogenes, Legionella pneumophila, Coxiella burnetii, Chlamydia trachomatis, and Helicobacter pylori (50–54). Downregulation of the citrate synthase GltA (and the citrate transporter NGFG_00249) by the sibling sRNAs (19, 21) might promote the channeling of glutamate-derived 2-oxoglutarate into the TCA cycle. It is interesting to note that *gltA* is also a target of Pasteurella multocida GcvB (38). In the sRNA double mutant, activity of the ED and PPP pathways was seemingly dampened, while the TCA cycle was more active, which is in accordance with the fact that several TCA cycle genes are under negative control of the sibling sRNAs (Table 1) (19, 21). Interestingly, expression of the ED pathway enzymes glucose 6-phosphate 1-dehydrogenase (*zwf*) and 6-phosphogluconolactonase (*pgl*) in meningococci was reported to be negatively regulated by the transcription factor GdhR (55), which itself is under negative control of the sibling sRNAs in N. gonorrhoeae (19). Furthermore, in meningococci, it has been shown that ED pathway genes are upregulated in the presence of glucose, while TCA cycle genes and *gdhR* are downregulated (56). However, *zwf* and *pgl* were not differentially expressed in a *gdhR* mutant of N. gonorrhoeae (26), and consistently, we did not detect changes in *zwf* mRNA amounts in the ΔΔ162/163 mutant by qRT-PCR (data not shown). Nevertheless, the labeling experiments clearly support the notion that the two putative modules of the bipartite metabolic network in N. gonorrhoeae are under opposite regulation by the sRNAs (Fig. 10e). This suggests a role for the sRNAs in optimizing growth of the pathogen while adapting to different environments during infection.

In this study, we demonstrate that the sibling sRNAs modulate BCAA (NGFG_00045)/phenylalanine (NGFG_01564) and glycine/alanine (NGFG_01721) import in a reciprocal manner (Fig. 5 and 10). Derepression of glycine import in the absence of NgncR_162/163 is concomitant with upregulation of *gcvH* and *glyA* (Fig. 2a, 6, and 10e), suggesting an important role for the sibling sRNAs in the regulation of serine-glycine metabolism, which in turn impacts the biosynthesis of nucleotides, vitamins, and other amino acids via the supply of C1 units. In fact, based on isotopologue profiling data, we propose that the threonine utilization (Tut) cycle (31, 57) is active in *Neisseria* to enable serine biosynthesis from threonine in the absence of 3-phosphoglycerate dehydrogenase, which is not encoded in the genome of the pathogenic *Neisseria* species. Both serine and threonine were efficiently taken up by wild-type gonococci during growth in chemically defined medium, while the glycine concentration remained almost unchanged (Fig. S6). In contrast, in the sRNA double mutant, the need for serine uptake was compensated to a certain extent by increased glycine uptake and cleavage via the glycine cleavage system, followed by a rise in serine biosynthesis from glycine and 5,10-MTHF due to upregulation of GlyA. This was accompanied by increased ^13^C enrichment in threonine, which was converted to glycine to a lesser extent (Fig. 10). The fact that threonine is a precursor to serine might even provide a hint for the biological relevance of positive regulation of the BCAA transporter NGFG_00045 by the sibling sRNAs: since threonine is also a precursor in the biosynthesis of isoleucine, increasing the uptake of isoleucine might spare threonine for use in serine synthesis.

Expression of lactoylglutathione lyase, also named glyoxalase I (GloA), is positively regulated by the sibling sRNAs (Table 1; Fig. 2a). The glyoxalase system mediates the detoxification of the highly reactive compound methylglyoxal, which is a by-product of glycolysis and gluconeogenesis during the conversion of triose phosphate isomers. First, methylglyoxal and glutathione are converted to *S*-lactoylglutathione by glyoxalase I, which is then cleaved by glyoxalase II, yielding d-lactate and glutathione (reviewed in reference 58). Interestingly, methylglyoxal is also formed during the catabolism of threonine via threonine dehydrogenase in the Tut cycle, due to the decarboxylation of α-amino-β-ketobutyrate and the subsequent oxidation of the intermediate aminoacetone (59). Upregulation of glyoxalase I is concomitant with downregulation of glycine uptake by the sibling sRNAs, which we propose to result in increased Tut cycle activity.

Other new members of the NgncR_162/163 regulon are related to the synthesis and maintenance of iron sulfur (Fe-S) centers (Table 1; Fig. 2a). DnrN is supposed to be involved in the repair of Fe-S centers damaged by oxidative or nitrosative stress (60). Interestingly, *dnrN* and two other positively regulated NgncR_162/163 targets, *norB* and *aniA*, were shown to be under the control of the same transcription factor, NsrR (61). IscR is the transcriptional regulator of the *iscRSUA* operon encoding enzymes for Fe-S cluster biosynthesis. IscR itself contains an [Fe2-S2] cluster, and holo-IscR was shown to directly repress expression of the *iscRSUA* operon in E. coli and pathogenic bacteria in order to maintain proper Fe-S cluster homeostasis (reviewed in reference 62). Both *dnrN* and *iscR* were deregulated in a Δ*hfq* mutant of N. meningitidis (25), indicating posttranscriptional regulation. Since *dnrN* and *iscR* are inversely regulated by the sibling sRNAs, with *dnrN* being activated and *iscR* being repressed, metabolic enzymes containing Fe-S clusters might be of particular importance under conditions in which the sibling sRNAs are abundant. Besides glucose and pyruvate, gonococci can use lactate as a carbon and energy source, since electrons from the oxidation of both l- and d-lactate feed directly into the respiratory chain (63). Interestingly, one of the two gonococcal l-lactate dehydrogenases (LutACB) contains Fe-S clusters (64, 65) and lactate uptake is indirectly controlled by the sibling sRNAs via GdhR, which is a repressor of the *lctP* gene encoding lactate permease (26). Lactate permease is considered a virulence factor of N. gonorrhoeae, since *lctP*-deficient mutants are attenuated in a murine model of lower genital tract infection (66). It should be noted that RNA-seq also suggested the positive regulation of the NGFG_02263 gene, encoding the ortholog of the meningococcal sole glucose transporter (67), by the sibling sRNAs. In N. meningitidis, deletion of *hfq* resulted in downregulation of the glucose transporter transcript, arguing in favor of direct or indirect sRNA-mediated expression control (25). However, the NGFG_02263 putative target could not be validated by qRT-PCR.

In conclusion, the work presented here expands our knowledge about the mechanisms of action of the sibling sRNAs NgncR_162/163 and their regulon. The data demonstrate that the siblings do not exhibit a complete functional redundancy and that they can act both as negative and positive regulators, thus applying different mechanisms of action, which, however, need to be characterized in more detail in the future. Moreover, the combined results of RNA-seq analysis and isotopologue profiling point to the operation of a bipartite central carbon metabolism in N. gonorrhoeae and a role of the sibling sRNAs in the regulatory networks which govern these central metabolic pathways and link them to the requirement of nutrient uptake and, in particular, amino acid uptake. Thus, the sibling sRNAs appear to play a superior role in the regulatory hierarchy of central metabolic pathways of the gonococcus.

## MATERIALS AND METHODS

### Bacterial strains and growth conditions.

The N. gonorrhoeae mutants used in this study were derived from wild-type strain MS11 (GenBank accession number NC_022240.1) and are listed in Table S1 in the supplemental material. N. gonorrhoeae was grown on GC agar (Oxoid) plates with 1% vitamin mix (19) for 14 to 16 h at 37°C in a humidified 5% CO_2_ atmosphere. Liquid cultures were grown in PPM (Per 1 l of dH_2_O: proteose peptone no. 3 [15 g], soluble starch [1 g], KH_2_PO_4_ [4 g], K_2_HPO_4_ [1 g], NaCl [5 g]) containing 1% vitamin mix and 0.04% (wt/vol) NaHCO_3_. Growth in chemically defined medium was conducted in CDM10 (68) with slight modifications (l-glutamate, 0.0445 g/L; l-aspartate, 0.02 g/L). For metabolic labeling experiments l-proline and glucose were replaced by [U-^13^C_5_]l-proline (0.05 g/L; 0.4 mM) and [U-^13^C_6_]d-glucose (2.5 g/L; 13.9 mM), respectively. Bacteria were grown to an OD_550_ of 0.6 for 4 h in order to achieve steady-state conditions in protein-derived amino acids and fatty acids, which were subjected to isotopologue profiling. Media were supplemented with kanamycin, erythromycin, or spectinomycin at final concentrations of 40 μg/mL, 7 μg/mL, or 50 μg/mL, respectively, when required. Escherichia coli TOP10 (Thermo Fisher Scientific) and E. coli DH5α (69) were cultured in lysogeny broth (LB). When required, antibiotics were added to the following final concentrations: ampicillin, 100 μg/mL; kanamycin, 30 μg/mL; chloramphenicol, 30 μg/mL.

10.1128/mbio.03093-22.9 TABLE S1 Bacterial strains and plasmids used in this study. Download Table S1, DOCX file, 0.02 MB. Copyright © 2023 Steiner et al. 2023 Steiner et al. https://creativecommons.org/licenses/by/4.0/ This content is distributed under the terms of the Creative Commons Attribution 4.0 International license.

### Construction of N. gonorrhoeae mutants.

PCR primers for the amplification of DNA fragments used for mutant construction are listed in Table S2. For the synthesis of N. gonorrhoeae-specific fragments, chromosomal DNA of strain MS11 was used as the template. Clonings were performed in E. coli DH5α.

**(i) MS11 93gfp, MS11 ΔΔ93gfp, MS11 249gfp, and MS11 ΔΔ249gfp.** N. gonorrhoeae mutants carrying translational target-*gfp* fusions integrated into the intergenic region between the *iga* and *trpB* genes were obtained by transformation of strain MS11 or the ΔΔ162/163 mutant with DNA fragments composed of part of the *trpB* gene, the target-*gfp* fusion under the control of the promoter of the respective target gene, an erythromycin resistance cassette, and part of the *iga* gene. To generate appropriate DNA fragments, two consecutive steps of overlap extension PCR were performed. First, a 590-bp fragment from the 3′ end of the *trpB* gene (amplified with primer pairs trbB5/trpBF93 and trpB5/trpBF249) was combined with DNA fragments comprising the upstream region and 23 or 8 NGFG_00093 or NGFG_00249 codons, respectively, which were amplified using primer pairs 93up-5/93up-3 and 249up-5 and 249up-3. Subsequently, the resulting fragments were combined with a segment comprising *gfp*, *ermC*, and *iga* DNA, which was amplified from chromosomal DNA of mutant MS11 1721-*gfp* (19) with primer pair iga5/Lgfp5.

**(ii) MS11 gcvH-F and MS11 ΔΔgcvH-F.** In mutants MS11 gcvH-F and MS11 ΔΔgcvH-F, a C-terminal FLAG tag was added to *gcvH* via allelic-exchange mutagenesis. An appropriate DNA fragment for the transformation of N. gonorrhoeae MS11 and MS11 ΔΔ162/163 was obtained via overlap extension PCR by a combination of DNA segments comprising *gcvH* (amplified with primer pair gcvH-F1/gcvH-F2), the sequence encoding 3×FLAG followed by *ermC* (amplified with primer pair gcvH-3/gcvH-4), and the intergenic region between *gcvH* and the NGFG_01515 gene as well as part of the NGFG_01515 gene (amplified with primer pair gcvH-5/gcvH-6).

**(iii) MS11 P*_opa_*45, MS11 ΔΔP*_opa_*45 and MS11 ΔΔP*_opa_*45c.** A 648-bp DNA fragment from the upstream region of NGFG_00045 was amplified using primer pair 45-5UTR-1/45-5UTR-23 and was cloned into vector plasmid pSL1180 (70) together with an erythromycin resistance cassette amplified with primers 45-5ermC-13/45-5ermC-23 from plasmid pMR68 (71). The cloned DNA segments were then amplified with the outer primers 45-5UTR-1/ermPopa1. The *opa* promoter was PCR amplified with primer pair ermPopa2/45Popa-4 and combined with a DNA fragment comprising the 5′ UTR (24) and 546 bp of the NGFG_00045 coding region (amplified with primer pair 45Popa-5/45Flag-6) by overlap extension PCR. Finally, the DNA segments comprising the NGFG_00045 upstream region followed by *ermC* and the P*_opa_*-NGFG_00045 fusion were combined by overlap extension PCR (using primers 45-5UTR-1/45Flag-6), and the resulting DNA fragment was transformed into N. gonorrhoeae MS11 and ΔΔ162/163 (19) to yield strains MS11 P*_opa_*45 and MS11 ΔΔP*_opa_*45. For complementation, MS11 ΔΔP*_opa_*45 was transformed with plasmid pMR-162/163 (19) to yield strain MS11 ΔΔP*_opa_*45c.

**(iv) MS11 P_45_gfp and MS11 ΔΔP_45_gfp.** The fusion of the NGFG_00045 upstream region, including the 5′ UTR, to the *gfp*-mut2 gene was constructed by combining DNA fragments amplified with primer pairs 45gfp-1/45gfp-3 and 45gfp-2/45gfp-7 via overlap extension PCR. Plasmid pKEN (72) was used as the template for the amplification of *gfp*-mut2. The resulting DNA fragment was combined with a DNA fragment comprising *ermC* and 500 bp from the NGFG_00045 downstream region, which was obtained by overlap extension PCR using DNA fragments amplified with primer pairs 45-5ermC-13/45-3ermC-23 and 45gfp-6/45mut-5, respectively. Transformation of the resulting DNA fragment into N. gonorrhoeae MS11 and MS11 ΔΔ162/163 yielded strains MS11 P_45_gfp and MS11 ΔΔP_45_gfp, in which the NGFG_00045 gene is replaced by *gfp*. A consensus Shine-Dalgarno sequence was introduced in the NGFG_00045 5′ UTR by performing overlap extension PCR using DNA fragments amplified with primer pairs 45gfp-1/45gfp-8 and 45gfp-9/45mut-5 from chromosomal DNA of strain MS11 P_45_gfp. The combined DNA fragment was transformed into N. gonorrhoeae MS11 and MS11 ΔΔ162/163 to yield strains MS11 P_45_gfpSD and MS11 ΔΔP_45_gfpSD.

**(v) MS11 Δ45 and MS11 Δc45.** To create the Δ45 mutant, a DNA segment covering 289 bp from the upstream region and the sequence encoding the first 80 amino acids of NGFG_00045 was replaced by an erythromycin resistance cassette via allelic-exchange mutagenesis. In the DNA fragment used for transformation of MS11, the *ermC* gene (amplified with primer pair 45-5ermC-13/45-3ermC-3) is flanked by sequences comprising the NGFG_00044 3′ end and part of the intergenic region between the NGFG_00044 and NGFG_00045 genes (amplified with primer pair 45-5UTR-1/45-5UTR-23) and encoding amino acids 81 to 239 of NGFG_00045 (amplified with primer pair D451/D45-2). For complementation, overlap extension PCR was applied to insert a kanamycin resistance cassette (amplified with primer pair D45-4/D45-5) between the upstream fragment used for construction of the Δ45 mutant (amplified with primer pair 45-5UTR-1/D45-3) and a DNA segment covering the promoter region, the 5′ UTR, and 785 bp from the 5′ end of the NGFG_00045 gene (amplified with primer pair D45-5/D45-6). The resulting DNA fragment was transformed into MS11 Δ45 to yield MS11 Δc45.

**(vi) MS11 Δ1564.** In the Δ1564 mutant, the region encoding amino acids 1 to 243 of NGFG_01564 was replaced by an erythromycin resistance cassette. A DNA fragment comprising segments derived from the upstream region (PCR amplified with primer pair D1564-1/D1564-2) and the coding region of NGFG_01564 (covering amino acids 245 to 412, amplified with primer pair D1564-5/D1564-6), which flank the *ermC* gene (amplified with primer pair D1564-3/D1564-4), was assembled by overlap extension PCR and transformed into MS11. Homologous recombination yielded strain MS11 Δ1564.

**(vii) MS11 Δ1721.** In the Δ1721 mutant, the NGFG_01721 ORF as well as 248 bp from its upstream region was replaced by a spectinomycin resistance cassette. To construct this mutant, a SacI/PstI fragment comprising part of the upstream NGFG_01720 ORF and the intergenic region was amplified with primer pair 1721up1/1721up2 and was cloned together with a spectinomycin resistance cassette into pSL1180. The spectinomycin cassette expressing *aadA1* under the control of the *Neisseria* P*_opa_* promoter was amplified from MS11 ΔtfpR2 (73) using primer pair spec2(PstI)/Popa5(KpnI). The assembled fragments were subsequently amplified with primer pair 1721up1/D1721-4 and were combined via overlap extension PCR with a DNA fragment derived from the downstream region of NGFG_01721 (amplified with primer pair D1721-3/D1721-2). Transformation of MS11 with the full-length DNA fragment yielded strain MS11 Δ1721.

### Construction of plasmids for sRNA target validation in E. coli.

Validation of sRNA-target interactions was performed in E. coli using a GFP-SF-based reporter system (28). DNA fragments comprising the 5′ UTR and the first 8 to 32 codons of NGFG_00249, NGFG_00093, NGFG_01937, and *dnrN* targets were amplified with appropriate primer pairs (Table S1) and were cloned into the BfrBI- and NheI-digested plasmid pXG10-SF (28) to yield plasmids pXG-249, pXG-93, pXG-1937, and pXG-1146, respectively. Forward primers correspond to the annotated transcriptional start site in the case of NGFG_00093 and NGFG_01937 (24) or were chosen arbitrarily when the 5′ end of the transcript had not been mapped. Plasmid pXG-863 carrying a *glyA*-*gfp* fusion was obtained by cloning a DNA fragment comprising the intergenic region between NGFG_00864 and *glyA* and encoding the last 8 amino acids of NGFG_00864 as well as the first 40 codons of *glyA* into the intercistronic fusion vector pXG30-SF (28). Plasmid pXG-1722 carrying a *dadA*-*gfp* fusion was constructed accordingly via ligation of a DNA fragment comprising the intergenic region between NGFG_01721 and *dadA* and encoding the last 24 amino acids of NGFG_01721 as well as the first 40 codons of *dadA*. Plasmid pJV-162 expressing NgncR_162 and plasmid pJV-162m1 expressing the sRNA with a mutated SL2 sequence have been described previously (19).

### RNA preparation, RNA-seq, and analysis of RNA-seq data.

N. gonorrhoeae MS11 and MS11 ΔΔ162/163 were grown to an OD_550_ of 0.5 in PPM. RNA was prepared using the miRNeasy micro kit (Qiagen) according to the manufacturer’s instructions, followed by DNase I treatment. RNA integrity was checked using a Bioanalyzer (Agilent). After enrichment of mRNA using the Universal RiboDepletion kit (siTOOLs Biotech), cDNA preparation was performed with the NEBNext Ultra directional library preparation kit for Illumina (NEB). The cDNA was sequenced on a HiSeq 3000 (Illumina), yielding 100-bp paired-end reads. Reads with a minimum length of 15 bp after removal of low-quality ends and adapters using Cutadapt (74) were mapped to the N. gonorrhoeae MS11 genome (genome assembly ASM15685v2) (75). Read mapping was conducted using Bowtie2 (76). Genes were quantified using featureCounts (77). DESeq2 (22) was used to identify differentially regulated transcripts.

### Northern Blot analysis and real-time quantitative PCR.

Northern blot analysis was performed as described previously (19). Quantification of signal intensities was performed using ImageJ (78). For real-time quantitative PCR (qRT-PCR) experiments, 1 μg of RNase-free DNase-treated RNA was reverse transcribed with random hexamer primers using a RevertAid first-strand cDNA synthesis kit (Thermo Scientific). All qRT-PCRs were performed in triplicate in a 20-μL mixture containing cDNA (5 μL of 1:20 dilution), PerfeCTa SYBR green FastMix containing ROX reference dye (Quanta Biosciences), and 18 pmol of primer (Table S2). Amplification and detection of PCR products were performed with a StepOne Plus qRT-PCR system (Applied Biosystems) using the following procedure: 95°C for 10 min and then 40 cycles of 95°C for 15 s and 60°C for 60 s, followed by dissociation curve analysis. The relative expression levels of the genes studied were normalized to the 5S rRNA gene. Data were analyzed using the ΔΔ*C_T_* method (79). If not stated otherwise, at least three qRT-PCR experiments were performed in triplicate with cDNA that was reverse transcribed from independent RNA preparations.

### Immunoblot analysis.

For the analysis of GFP expression in E. coli, bacteria were grown to an OD_600_ of 1.0 in LB. Bacteria from a culture volume of 2 mL were pelleted and resuspended in 200 μL of Laemmli buffer. N. gonorrhoeae was grown to an OD_550_ of 0.5 in PPM. Cells from 1 mL of culture were harvested by centrifugation and resuspended in 50 μL of Laemmli buffer. Samples were incubated for 5 min at 95°C. Western blot analysis of the samples was performed as described previously (19). Quantification of signal intensities was performed using ImageJ (78).

### Analysis of amino acid composition of (spent) culture medium.

Amino acids in bacterial culture medium were measured by UPLC–electrospray ionization-tandem mass spectometry (UPLC–ESI–MS/MS) using an Acquity UPLC combined with a Quattro Premier triple-quadrupole mass spectrometer (Waters, Milford, MA, USA). Derivatization, chromatographic separation, and successive detection of amino acids were carried out as described by Salazar et al. (80), with modifications.

After sterile filtration, 100 μL of medium was diluted with 100 μL of methanol containing norvaline as an internal standard, with a final concentration of 1 mM. Twenty microliters of this mixture was used for subsequent amino acid derivatization using the AccQ·Tag Ultra derivatization kit (Waters, Milford, MA, USA) in accordance with the manufacturer’s instructions.

Chromatographic separation was carried out using an ethylene-bridged hybrid (BEH) C_18_ column (2.1 by 100 mm, 1.7-μm particle size; Waters) equipped with a VanGuard precolumn and an in-line particle filter. Elution was performed with 100% eluent A for 1 min, followed by a binary solvent gradient to 30% of eluent B within 12 min at a flow rate of 0.4 mL/min. Eluent A consisted of 0.1% formic acid in water, and eluent B consisted of 0.1% formic acid in acetonitrile.

The ESI source was operated in the positive mode at a source temperature of 120°C with the capillary voltage set to 3 kV, the cone voltage at 30 V, and the desolvation gas at 850 L/h at 400°C. Compounds were detected by multiple reaction monitoring (MRM; [M+H]^+^ → *m/z* 171) with a dwell time of 25 ms and a collision energy of 18 V and using argon as the collision gas at a flow rate of 0.3 mL/min. Data acquisition and processing were carried out using MassLynx and QuanLynx (Waters, Milford, MA, USA; version 4.1).

### Sample preparation for ^13^C analysis of protein-bound amino acids.

The analysis of protein-bound amino acids was done as previously described (81). About 1 mg of lyophilized bacterial cell pellet was hydrolyzed overnight at 105°C after the addition of 500 μL HCl (6 M). The hydrolysate was dried under a gentle stream of nitrogen at 70°C and dissolved in 200 μL of acetic acid (50%). For the isolation of protein-bound amino acids, a cation exchange column of Dowex 50WX8 (H^+^ form; 7 by 10 mm; 200 to 400 mesh, 34 to 74 μm) was washed with 1,000 μL of methanol (70%) and 1,000 μL of H_2_O (double distilled). After application of the sample, which was dissolved in acetic acid, to the column, the column was first evolved with 1,600 μL of H_2_O (bidest.). Subsequently, the amino acids were eluted with 1,000 μL of aqueous ammonia solution (4 M). After the ammonia eluate was dried under a gentle stream of nitrogen at 70°C, the isolated amino acids were incubated with 50 μL of *N*-methyl-*N*-*tert*-butyldimethylsilyltrifluoroacetamide (MTBSTFA) containing 1% *tert*-butyldimethylchlorsilane and 50 μL acetonitrile (anhydrous) for 30 min at 70°C. The *N*-*tert*-butyldimethylsilyl (TBDMS) derivates of the amino acids were analyzed by GC-MS.

### Sample preparation for ^13^C analysis of fatty acids.

The analysis of fatty acids was done as previously described (82). In brief, about 5 mg of the lyophilized bacterial cell pellet was dissolved in 1 mL of cold methanol and 800 mg of glass beads was added. Cells were mechanically disrupted and lysed using a Ribolyser system (Hybaid) with three cycles of 20 s at 6.5 m s^−1^. Then, the samples were centrifuged for 10 min at 7,000 rpm, and the supernatant was subsequently dried under a gentle stream of nitrogen at room temperature. For derivatization, the dry residue was incubated with 50 μL of MTBSTFA containing 1% *tert*-butyldimethylchlorsilane and 50 μL acetonitrile (anhydrous) for 1 h at 70°C. The TBDMS metabolites were analyzed by GC-MS.

### GC-MS measurement parameters.

For the analysis of TBDMS-amino acids, a QP2010 Plus gas chromatograph-mass spectrometer was used as previously described (80). The column was heated to 150°C, kept at 150°C for 3 min, heated to 280°C with a temperature gradient of 7°C min^−1^, and kept at 280° for 3 min. For analysis of TBDMS-fatty acids (52), the column was kept at 100°C for 2 min and subsequently heated to 234°C (3°C min^−1^). The column was then heated at 1°C min^−1^ to 237°C. Finally, the column was heated to 260°C (3°C min^−1^).

Each sample was measured in triplicate in order to account for technical errors. GC-MS data were processed with Shimadzu LabSolution software v4.20. For the calculation of ^13^C excess values and isotopologue profiles, Isotopo software was used (34).

### Data availability.

RNA-seq data obtained in this study have been deposited in the GEO database under accession number GSE177032.
