# Supplementary figures and images for: Central Role of Sibling Small RNAs NgncR_162 and NgncR_163 in Main Metabolic Pathways of Neisseria gonorrhoeae
Source: mBio. 2023 Jan 4;14(1):e03093-22. doi: 10.1128/mbio.03093-22 (PMC9973317; doi:10.1128/mbio.03093-22)

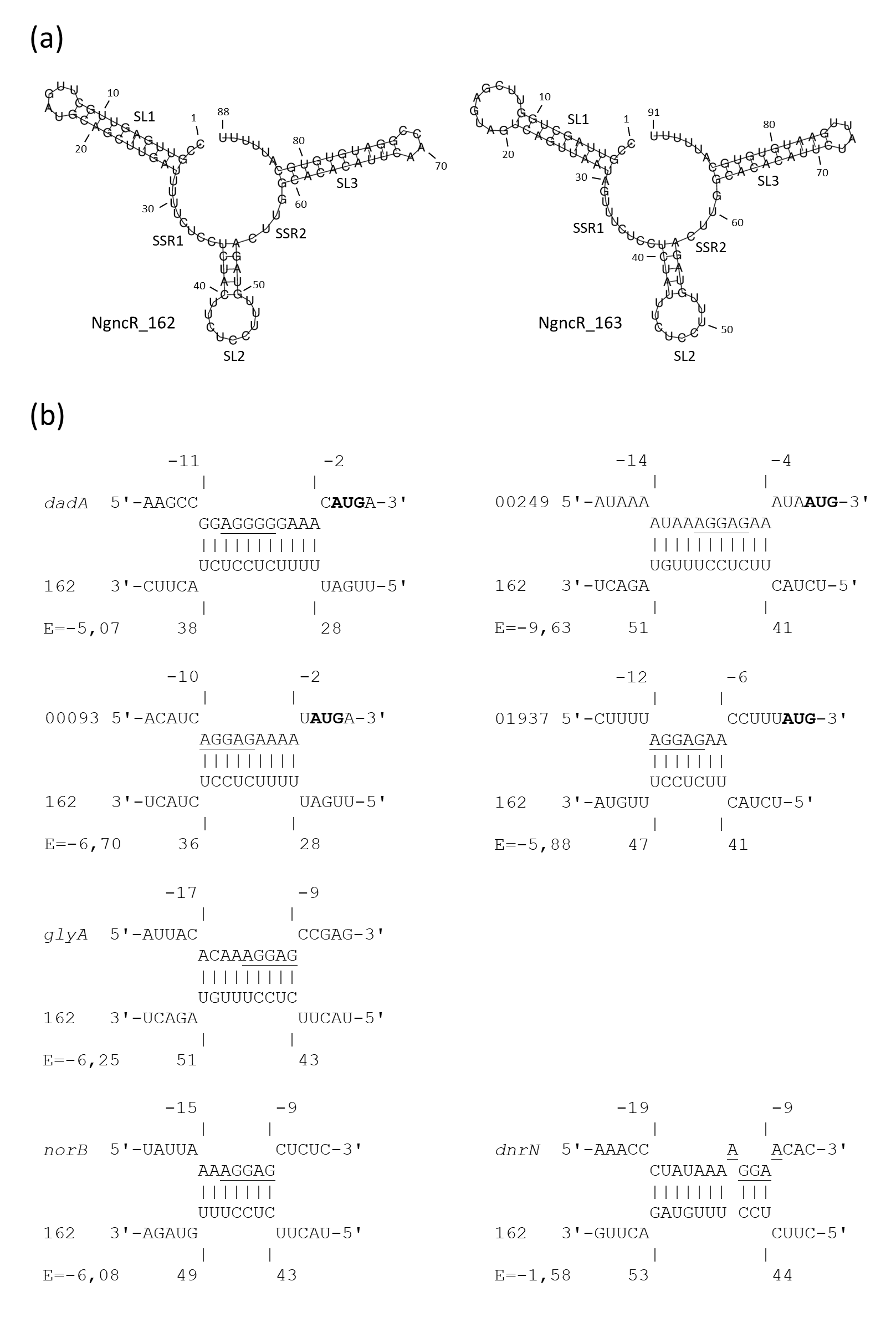

Supplement: FIG S1 [file mbio.03093-22-s0001.tif]

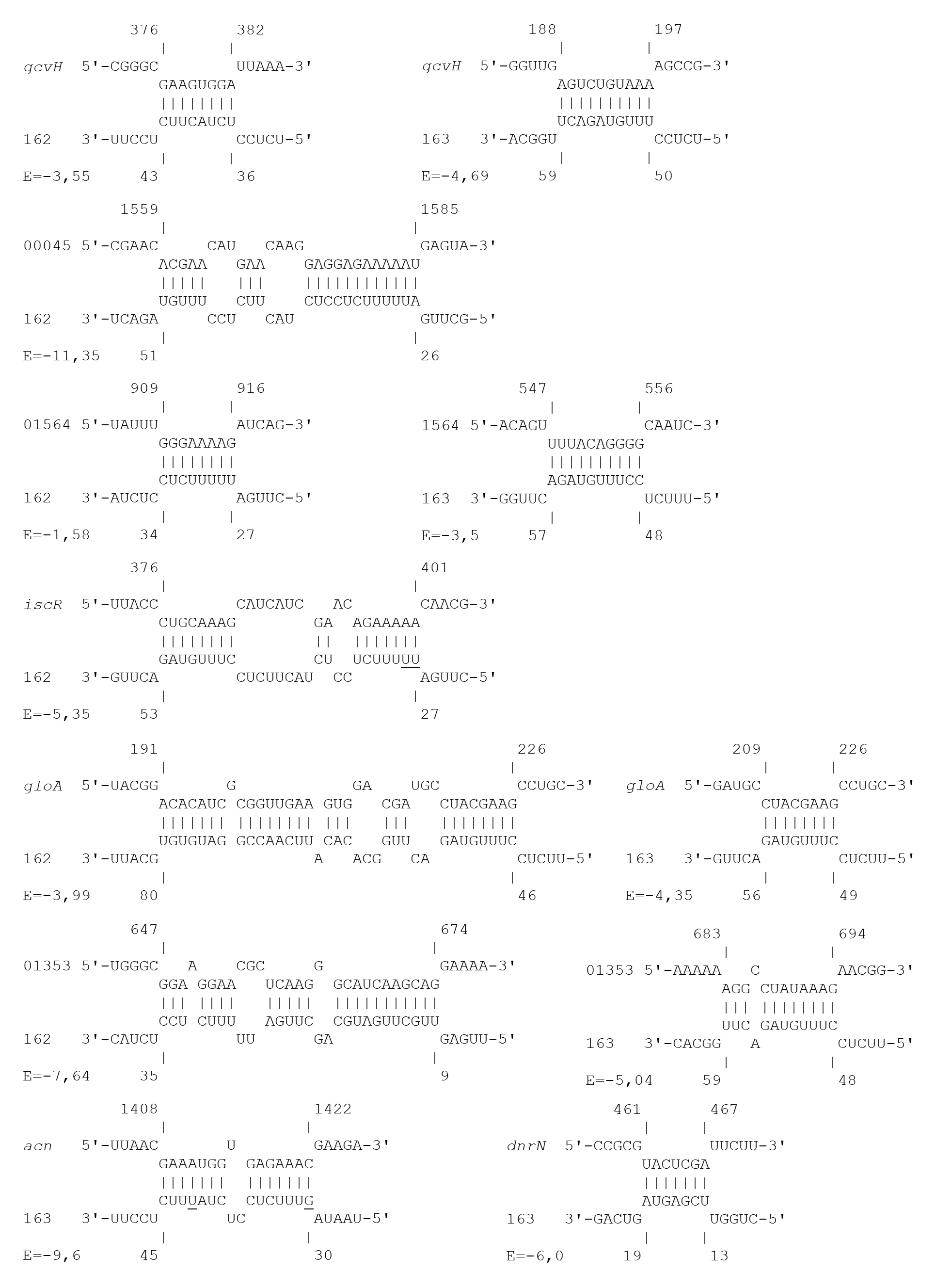

Supplement: FIG S2 [file mbio.03093-22-s0002.tif]

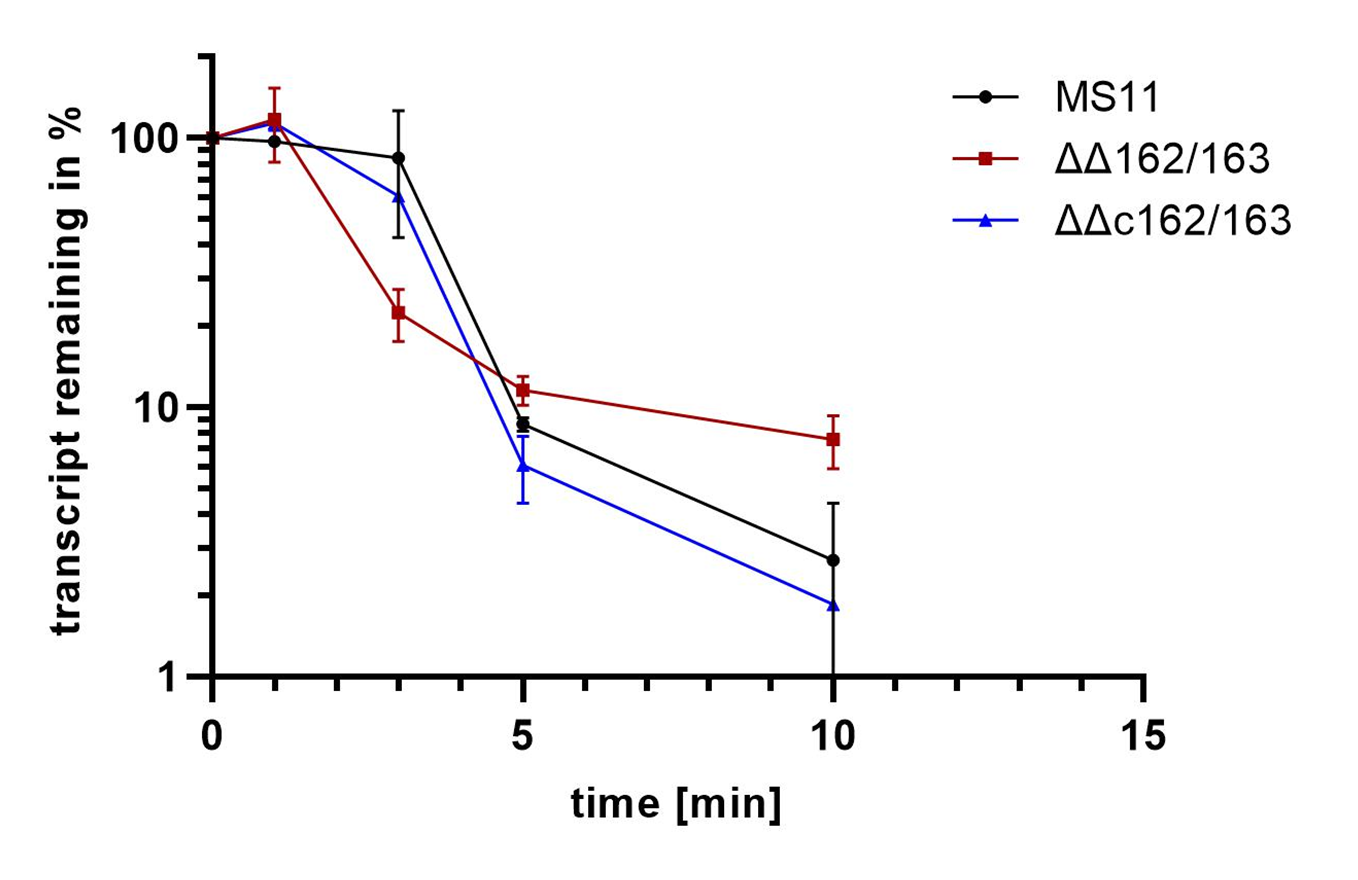

Supplement: FIG S3 [file mbio.03093-22-s0003.tif]

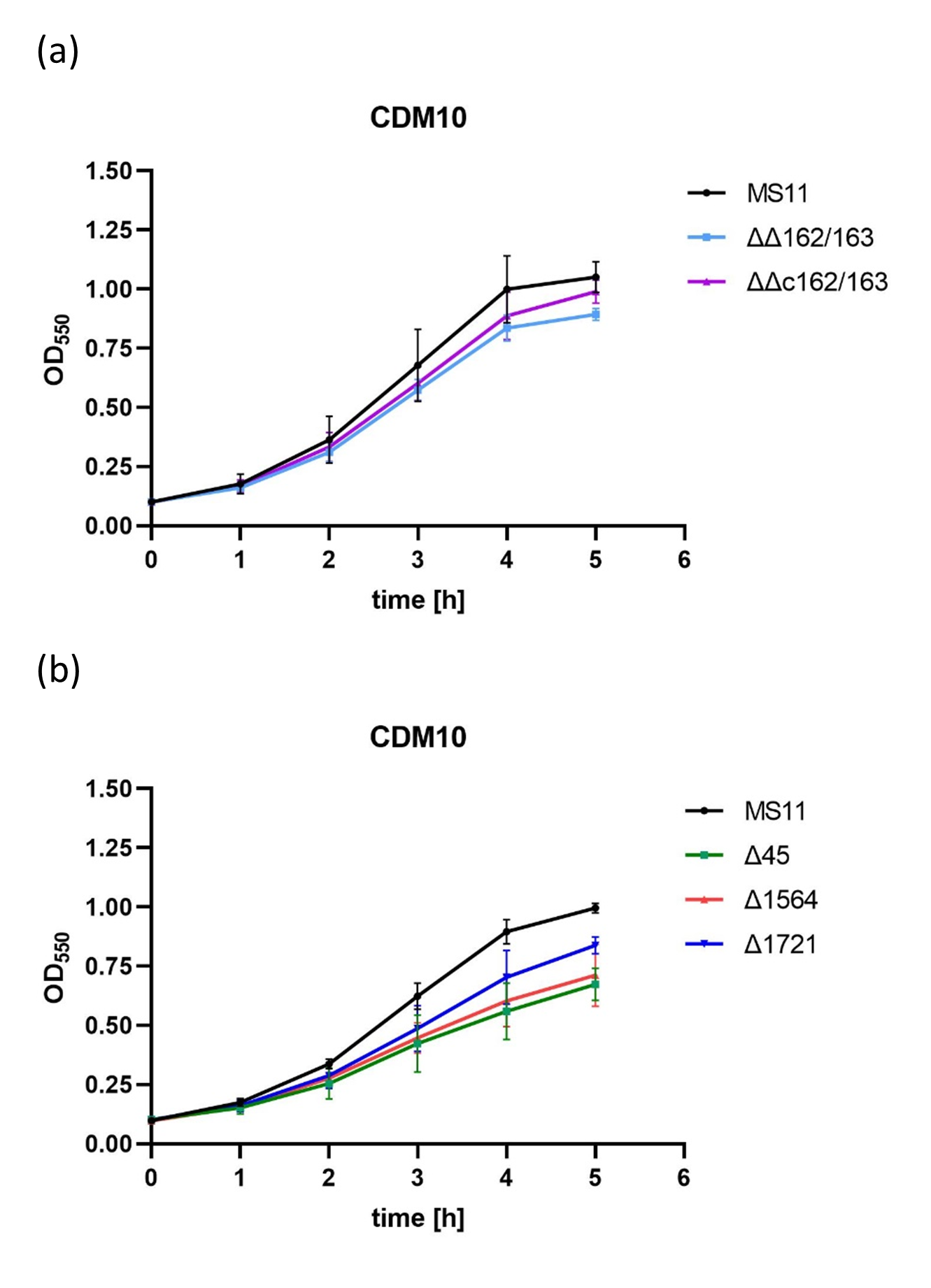

Supplement: FIG S4 [file mbio.03093-22-s0004.tif]

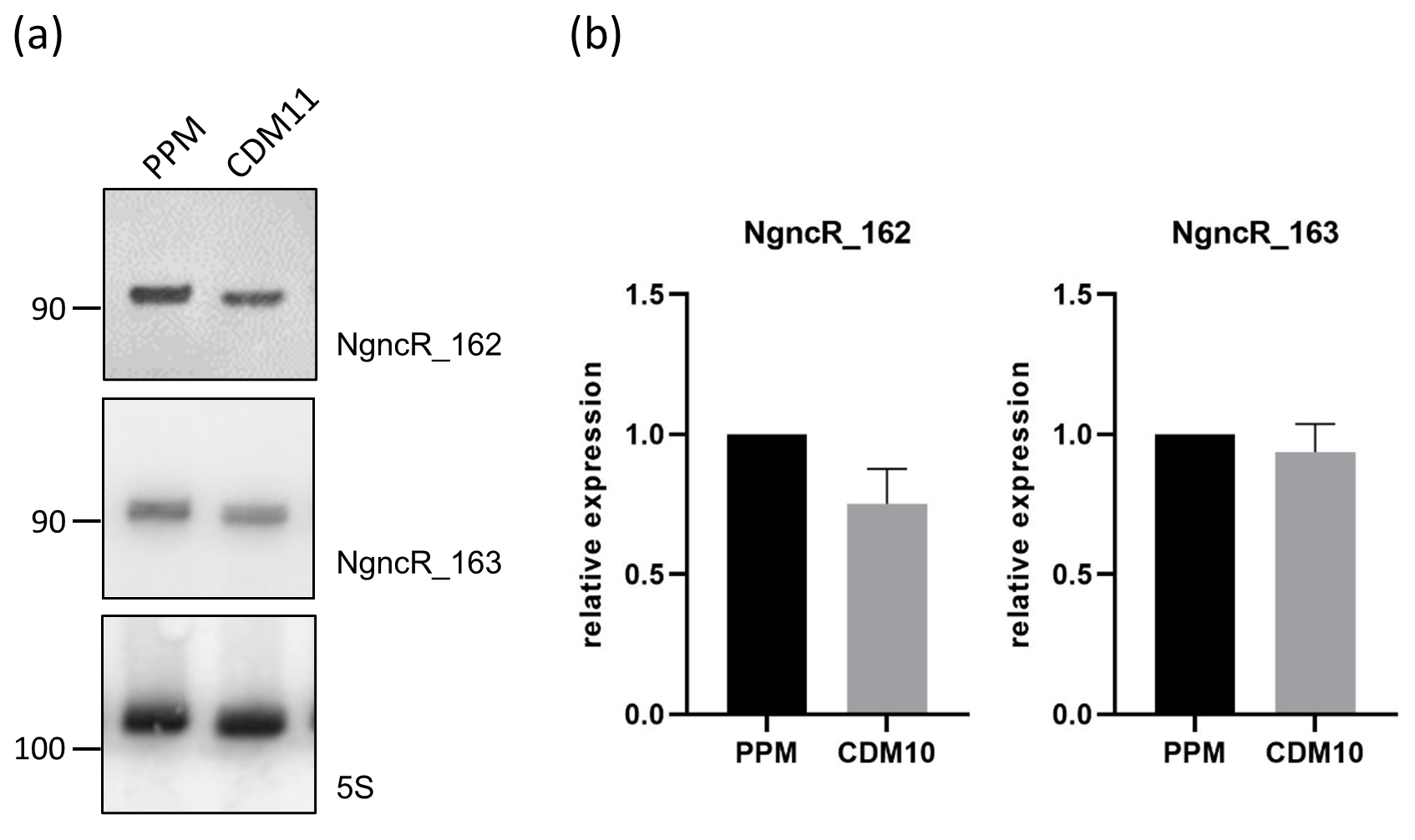

Supplement: FIG S5 [file mbio.03093-22-s0005.tif]

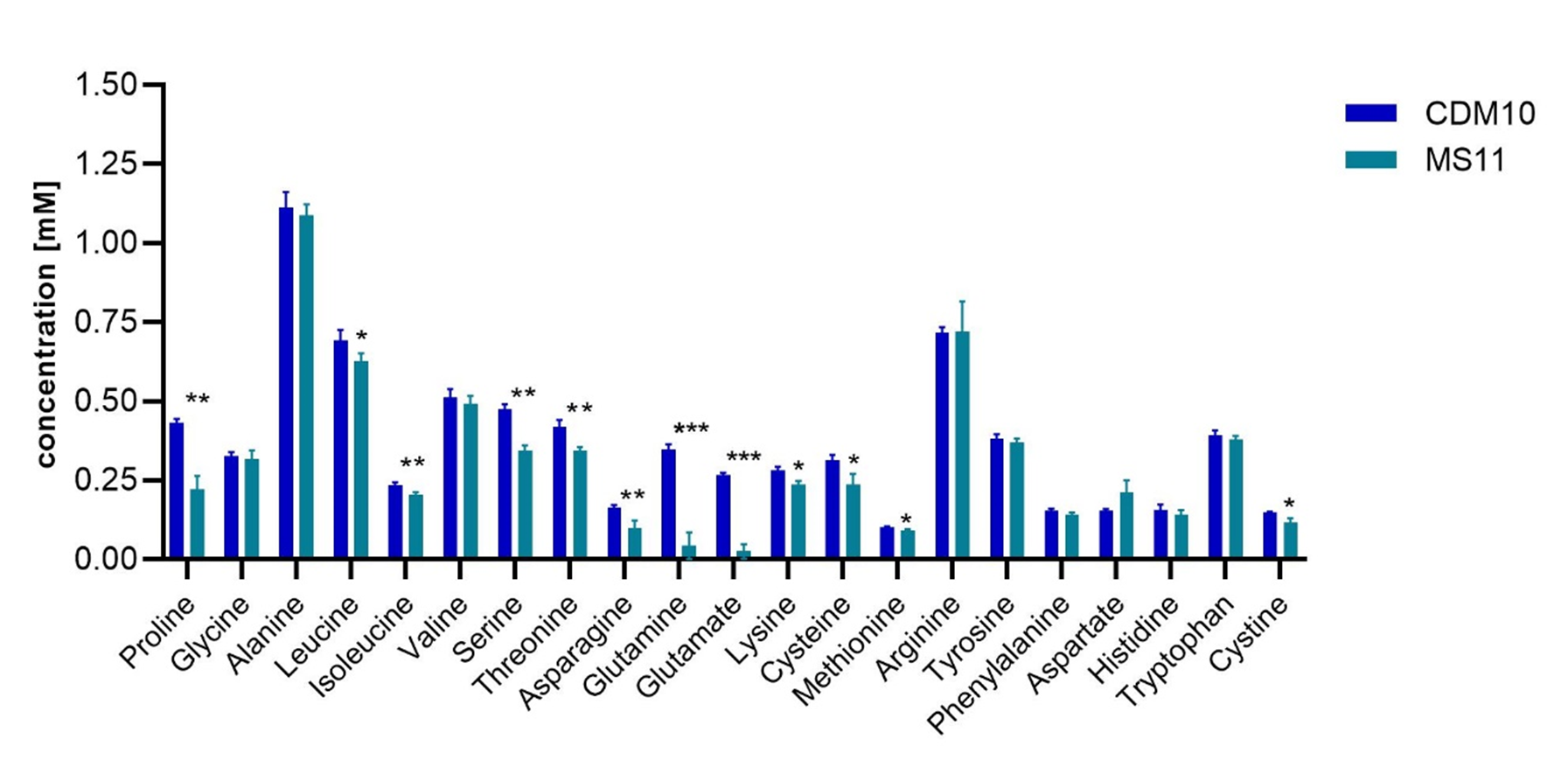

Supplement: FIG S6 [file mbio.03093-22-s0006.tif]

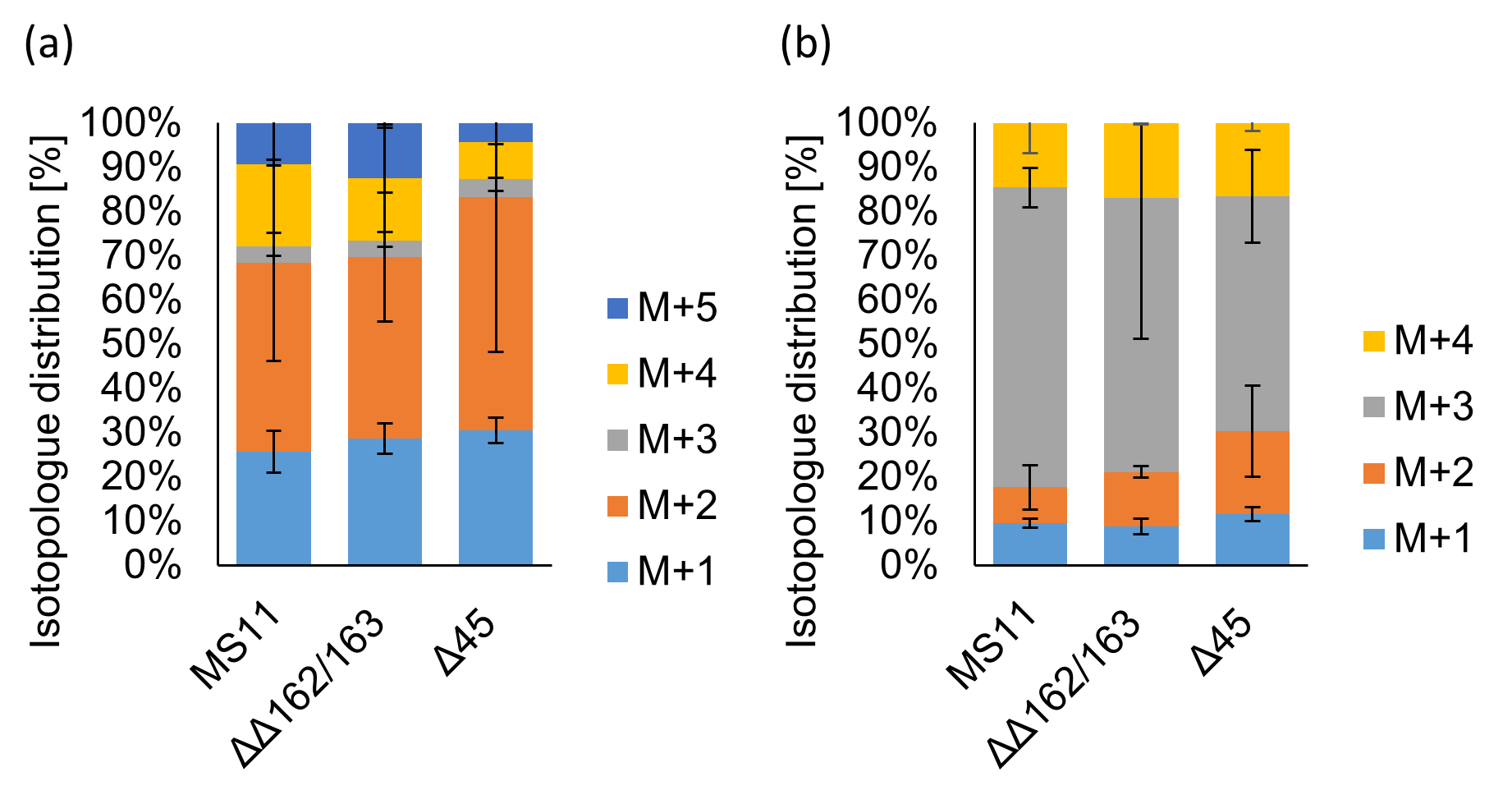

Supplement: FIG S7 [file mbio.03093-22-s0007.tif]

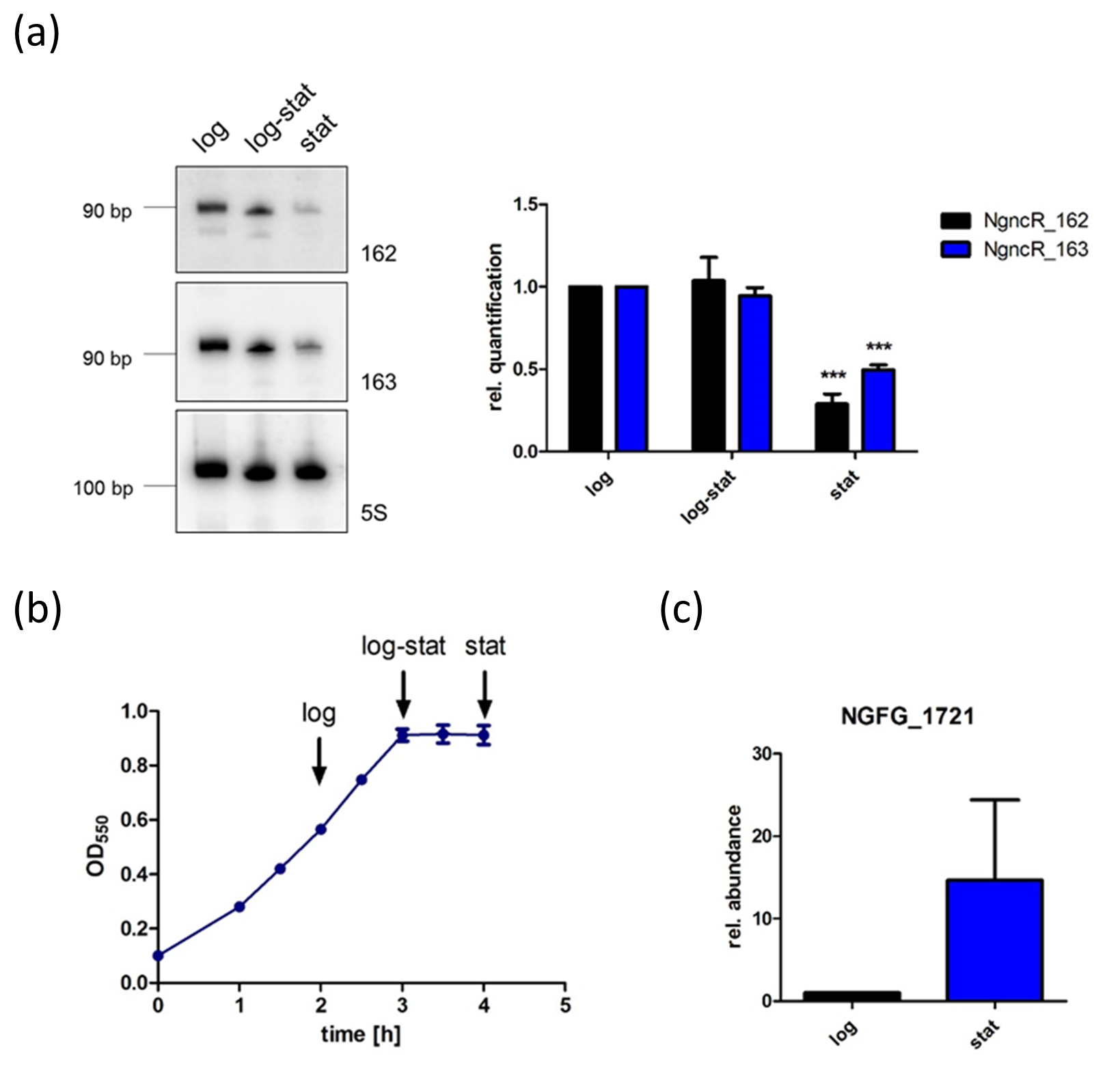

Supplement: FIG S8 [file mbio.03093-22-s0008.tif]
