# Supplementary material for: Central Role of Sibling Small RNAs NgncR_162 and NgncR_163 in Main Metabolic Pathways of Neisseria gonorrhoeae
Source: mBio. 2023 Jan 4;14(1):e03093-22. doi: 10.1128/mbio.03093-22 (PMC9973317; doi:10.1128/mbio.03093-22)
Supplement: TABLE S2 [file mbio.03093-22-s0010.docx]

**Tab. S2:** **Oligonuncleotides used in this study**

| Name | Sequence (5‘ to 3‘)^a^ | amplification of |
| --- | --- | --- |
| trpB5 | AGGAAGCCACCGGCAGACA | part of *trpB* |
| trpF249 | CGCAAAACCCGAACCGGTT GTCGACCTCGACTCATAGCAA | part of *trpB* with 3’-overhang to upstream region of NGFG_00249 |
| 249up-5 | TTGCTATGAGTCGAGGTCGAC AACCGGTTCGGGTTTTGCGG | upstream region and first 8 codons of NGFG_00249 with 5’-overhang to *trpB* |
| 249up-3 | gaattcgccagaaccagcagcggagcc CAGCAATCCGATAAACGTCAGC | upstream region and first 8 codons of NGFG_00249 with 3’-overhang to *gfp* |
| Lgfp-5 | GGCTCCGCTGCTGGTTCTGGCGAATTC ATGAGTAAAGGAGAAGAACTTTTCA | *gfp*-SF |
| iga5 | TGCGGGCAGCTTGAGAAGC | part of *iga* |
| trpF93 | CGCTGTATGACAAAAGCCCGA GTCGACCTCGACTCATAGCAA | part of *trpB* with 3’-overhang to upstream region of NGFG_00093 |
| 93up-5 | TTGCTATGAGTCGAGGTCGAC TCGGGCTTTTGTCATACAGCG | upstream region and first 23 codons of NGFG_00093 with 5’-overhang to *trpB* |
| 93up-3 | gaattcgccagaaccagcagcggagcc TGCGGCGGCGGAAAGGGTT | upstream region and first 23 codons of NGFG_00093 with 3’-overhang to *gfp* |
| gcvH-F1 | TTCCGCAGCAGAAACTGCCTTCCGCCCAAAAGC | *gcvH* |
| gcvH-F2 | CACCGTCATGGTCTTTGTAGTC ATCCACTTCGCCCGCGTATTG | *gcvH* with 3’-overhang to 3xFLAG sequence |
| gcvH-F3 | CAATACGCGGGCGAAGTGGAT GACTACAAAGACCATGACGGTG | 3xFLAG and *ermC* with 5’-overhang to *gcvH* |
| gcvH-F4 | CGTCGGACAAACGGGCGGT CACGAAAAACAAGTTAAGGGATGC | 3xFLAG and *ermC* with 3’-overhang to intergenic region between *gcvH* and NGFG_01515 |
| gcvH-F5 | GCATCCCTTAACTTGTTTTTCGTG ACCGCCCGTTTGTCCGACG | upstream region and part of NGFG_01515 with 5’-overhang to *ermC* |
| gcvH-F6 | CATACTTTCCTGTTCTTGAGCC | upstream region and part of NGFG_01515 |
| 45-5UTR-1 | taatgaattcgccgtgctgaaCTCCGCTGCGCC  AAATCGTTGCC | 3’-end of NGFG_00044 and part of intergenic region between NGFG_00044 and NGFG_00045 |
| 45-5UTR-23 | CAATTAACCCTCACTAAAggtaccGG  AAATAACCGAAACCGGACG | 3’-end of NGFG_00044 and part of intergenic region between NGFG_00044 and NGFG_00045 with 3’-overhang to *ermC* |
| 45-5ermC-13 | CGTCCGGTTTCGGTTATTTCCggtacc  TTTAGTGAGGGTTAATTG | *ermC* with 5’-overhang to upstream region of NGFG_00045 |
| 45-5ermC-23 | CCTTAACAGGGAAAGCAGCAGctgcag  GTACACGAAAAACAAGTT | *ermC* with 3’-overhang to downstream region of NGFG_00045 |
| ermPopa1 | TGGCGGATTAACAAAAACCGGctgcag  GTACACGAAAAACAAGTT | *ermC* with 3’-overhang to *opa* promoter (P*_opa_*) |
| ermPopa2 | CTTGTTTTTCGTGTACCTGCAG  CCGGTTTTTGTTAATCCGCCA | P*_opa_* with 5’-overhang to *ermC* |
| 45Popa-4 | CAATCTATGTGCTTATCGTAAAAA  ATTATATCGGGTTCCGGGCG | P*_opa_* with 3’-overhang to the 5’-UTR of NGFG_00045 |
| 45Popa-5 | CGCCCGGAACCCGATATAAT  TTTTTACGATAAGCACATAGATTG | 5’-UTR and part of coding region of NGFG_00045 with 5’-overhang to P*_opa_* |
| 45Flag-6 | attatagagctcGTAAAAACCCACACGCCCGCCAA | 5’-UTR and part of coding region of NGFG_00045 |
| 45gfp-1 | tataatgtcgacGCGAATAAGTGCGGCTAAGG | promoter region and 5’-UTR of NGFG_00045 |
| 45gfp-3 | GTGAAAAGTTCTTCTCCTTTACTCAT ATATAGAAACAGCGTCTGACAGG | promoter region and 5’-UTR of NGFG_00045 with 5’-overhang to *gfp*-mut2 |
| 45gfp-2 | CCTGTCAGACGCTGTTTCTATATATGAGT AAAGGAGAAGAACTTTTC | *gfp*-mut2 with 5’-overhang to 5’-UTR of NGFG_00045 |
| 45gfp-7 | CAATTAACCCTCACTAAAggtacctctagaGCC  GTCTGAAAACAGCC | *gfp*-mut2 with 3’-overhang to *ermC* |
| 45gfp-6 | GCTGTTTTCAGACGGCtctagaggtaccTTTAG  TGAGGGTTAATTG | downstream region of NGFG_00045 with 5’-overhang to *ermC* |
| 45mut-5 | ATTATAGAGCTCGGGGTGCAATATCTAAGG  AATT | downstream region of NGFG_00045 |
| 45gfp-8 | CCTTTACTCATATGTATATCTCCTTCTGAC AGGGATAATGTCTTC | promoter region and mutated 5’-UTR of NGFG_00045 with 5’-overhang to *gfp*-mut2 |
| 45gfp-9 | GAAGACATTATCCCTGTCAGAAGGAGA TATACATATGAGTAAA | *gfp*-mut2 with 5’-overhang to mutated 5’-UTR of NGFG_00045 |
| 45-3ermC-3 | CGATGGCATAATCGAGCAGCCTGCAGG TACACGAAAAACAAGTT | *ermC* with 3’-overhang to coding region of NGFG_00045 |
| D45-1 | CTTGTTTTTCGTGTACCTGCAGGCTGCT  CGATTATGCCATCG | coding region of NGFG_00045 with 5’-overhang to *ermC* |
| D45-2 | ACCATAATGCCGAAGCAGATGG | coding region of NGFG_00045 |
| D45-3 | TGAGACACAATTCATCGATGATGGAAAT AACCGAAACCGGACG | 3’-end of NGFG_00044 and part of intergenic region between NGFG_00044 and NGFG_00045 with 3’-overhang to kan^r^ |
| D45-4 | CGTCCGGTTTCGGTTATTTCCATCATCG ATGAATTGTGTCTCAA | kan^r^ with 5’-overhang to intergenic region between NGFG_00044 and NGFG_00045 |
| D45-5 | CCAACGCGGCAGGAATCTATCCTGAAG CTTGCATGCCTGCA | kan^r^ with 3’-overhang to upstream region of NGFG_00045 |
| D45-6 | TGCAGGCATGCAAGCTTCAGGATAGAT  TCCTGCCGCGTTGG | upstream region of NGFG_00045 with 5’-overhang to kan^r^ |
| D1564-1 | gccgtctgaaATTGAAGCCTGTGTGATACTGC | upstream region of NGFG_01564 |
| D1564-2 | CGCAATTAACCCTCACTAAAGAAGGCTT TCAGACGGCATAGG | upstream region of NGFG_01564 with 3’-overhang to *ermC* |
| D1564-3 | CCTATGCCGTCTGAAAGCCTTCTTTAGT GAGGGTTAATTGCG | *ermC* with 5’-overhang to upstream region of NGFG_01564 |
| D1564-4 | GGACAGCCAGCGCAATAAAGACGAAAA ACAAGTTAAGGGATGC | *ermC* with 3’-overhang to coding region of NGFG_01564 |
| D1564-5 | GCATCCCTTAACTTGTTTTTCGTCTTTATT GCGCTGGCTGTCC | part of the coding region of NGFG_01564 with 5’-overhang to *ermC* |
| D1564-6 | AAAATAGCCGTGCCGATAAGC | part of the coding region of NGFG_01564 |
| 1721up1 | tataatgagctcTTCCAATTCTGCCGCATCG | upstream region of NGFG_01721 |
| 1721up2 | tataatctgcagAATGCTGCCGACTGCCATACT | upstream region of NGFG_01721 |
| Spec2(PstI) | tataatctgcagTGTAGGGCTTATTATGCAGC | spectionomycin resistance cassette (spec^r^) |
| Popa5(KpnI) | tataatggtaccGGTTTTTGTTAATCCGCCA | spectionomycin resistance cassette (spec^r^) |
| D1721-4 | CGTGCCGTCTGAATATTCGggtaccGGTT  TTTGTTAATCCG | spectinomycin resistance cassette with 3’-overhang to downstream region of NGFG_01721 |
| D1721-3 | CGGATTAACAAAAACCggtaccCGAATA  TTCAGACGGCACGGC | downstream region of NGFG_01721 with 5’-overhang to spec^r^ |
| D1721-2 | tataatggatccGATGACCGTTACTTCATGTCC | downstream region of NGFG_01721 |
| 5UTR863-1 | tataatatgcat TTACGCATTATGGGCTATCTGC | intergenic region and regions encoding the last 8 amino acids (aa) of NGFG_00864 and first 40 aa of NGFG_00863 (*glyA*) |
| 5UTR863-2 | tataatgctagcGCTGACGTAGTTTTCAGAGGC | intergenic region and regions encoding the last 8 aa of NGFG_00864 and first 40 aa of NGFG_00863 (*glyA*) |
| 5UTR249-1 | tataatatgcatGTTTTGCCTGCACGGGCGG | 5’-UTR and region encoding the N-terminal 8 aa of NGFG_00249 |
| 5UTR249-2 | tataatgctagcCAGCAATCCGATAAACGTCAGC | 5’-UTR and region encoding the N-terminal 8 aa of NGFG_00249 |
| 5UTR93-1 | tataatatgcatCCCTGTTCGCGGCAAATGC | 5’-UTR and region encoding the N-terminal 23 aa of NGFG_00093 |
| 5UTR93-2 | tataatgctagcTTGACCGCCGCAGGCTGC | 5’-UTR and region encoding the N-terminal 23 aa of NGFG_00093 |
| 5UTR1937-1 | tataatatgcatTATTTTCATACAAAAAAACACCGC | 5’-UTR and region encoding the N-terminal 31 aa of NGFG_01937 |
| 5UTR1937-2 | tataatgctagcACGTTCCCACAATTCGATATGG | 5’-UTR and region encoding the N-terminal 31 aa of NGFG_01937 |
| 5UTR1146-1 | tataatatgcatGGTGTATGCTCCGTTTAAATAG | 5’-UTR and region encoding the N-terminal 32 aa of NGFG_01146 (*dnrN*) |
| 5UTR1146-2 | tataatgctagcTTCAAATTGTGCGCGGTGGAC | 5’-UTR and region encoding the N-terminal 32 aa of NGFG_01146 (*dnrN*) |
| 5UTR1722-1 | tataatatgcatTTGATGGCGAAAGTCATCGTC | region encoding the C-terminal 24 aa of NGFG_01721, intergenic region and region encoding the N-terminal 40 aa of NGFG_01722 (*dadA*) |
| 5UTR1722-2 | tataatgctagcACTGGTTTCCATCGCCACAC | region encoding the C-terminal 24 aa of NGFG_01721, intergenic region and region encoding the N-terminal 40 aa of NGFG_01722 (*dadA*) |
| 5S FW | CGGCCATAGCGAGTTGGT | amplicon for 5S RNA transcript quantification |
| 5S RV | TTGGCAGTGACCTACTTTCG | amplicon for 5S RNA transcript quantification and Northern Blot probe for 5S RNA |
| qRT45-1 | TCAGGACAAGCTGAACATCG | amplicon for NGFG_00045 transcript quantification |
| qRT45-2 | TTTGTCCATCACGTCCAAAA | amplicon for NGFG_00045 transcript quantification |
| qRT1146-1 | TGGCGCAACCGTTGATCATA | amplicon for NGFG_01146 *(dnrN)* transcript quantification |
| qRT1146-2 | GCAATTTCCGCCGGAAAGGT | amplicon for NGFG_01146 *(dnrN)* transcript quantification |
| qRT1163-1 | CCTCCCGCACAAATCAACAT | amplicon for NGFG_01163 (*icsR*) transcript quantification |
| qRT1163-2 | AATTCTCCCAAAGGTCGTGC | amplicon for NGFG_01163 (*icsR*) transcript quantification |
| qRT1353-1 | CAACGTCAACAGCTTCCTGA | amplicon for NGFG_01353 transcript quantification |
| qRT1353-2 | CGGTAAAGACCTGCATCCAT | amplicon for NGFG_01353 transcript quantification |
| qRT1407-1 | TACCACCTGTAACGGCATGA | amplicon for NGFG_01407 (*acn*) transcript quantification |
| qRT1407-2 | AGGAAAGCCTGTTTCGCATA | amplicon for NGFG_01407 (*acn*) transcript quantification |
| qRT1722-1 | CAATAAAGAGCGCATGGTCA | amplicon for NGFG_01722 (*dadA*) transcript quantification |
| qRT1722-2 | GCTTCGACTTCTTCGGTTTG | amplicon for NGFG_01722 (*dadA*) transcript quantification |
| qRT2111-1 | CAACTGGGATACGGAACGAT | amplicon for NGFG_02111 (*gloA*) transcript quantification |
| qRT2111-2 | GTTGTGCCGTGTTTCATCAG | amplicon for NGFG_02111 (*gloA*) transcript quantification |
| qRT2263-1 | GGCAAAGTCGGCTACAAAAA | amplicon for NGFG_02263 transcript quantification |
| qRT2263-2 | CCGGAAGCCAAAATAAACAA | amplicon for NGFG_02263 transcript quantification |
| qRT249-1 | CGATGATAGGCGGTTTGATT | amplicon for NGFG_00249 transcript quantification |
| qRT249-2 | CGACCGATAAAAACGTCGTC | amplicon for NGFG_00249 transcript quantification |
| qRT93-1 | TGGACATCAACGTCTTCCAA | amplicon for NGFG_00093 transcript quantification |
| qRT93-2 | GATTTCAGTTTGCCCGGATA | amplicon for NGFG_00093 transcript quantification |
| qRT1564-1 | ACCTCTTGGTTTCCCTGCTT | amplicon for NGFG_01564 transcript quantification |
| qRT1564-2 | GTACCGAACGGCATTTTCAT | amplicon for NGFG_01564 transcript quantification |
| qRT1937-1 | AGAAGCCGCCGATATTGATG | amplicon for NGFG_01937 transcript quantification |
| qRT1937-2 | TATTCATCGCATTGGGCAGC | amplicon for NGFG_01937 transcript quantification |
| qRT1471-1 | ATTGGCGATGGTCAACGAAG | amplicon for NGFG_01471 transcript quantification |
| qRT1471-2 | CCATTCTTCTTTGCTGGTCAAA | amplicon for NGFG_01471 transcript quantification |
| qRT1514-1 | CCGTCGGTATTACCCATCAC | amplicon for NGFG_01514 (*gcvH*) transcript quantification |
| qRT1514-2 | TGCGGCTTTTACATACTCAA | amplicon for NGFG_01514 (*gcvH*) transcript quantification |
| qRT2153-1 | GCCCAACATAAAGATGGCGG | amplicon for NGFG_02153 (*norB*) transcript quantification |
| qRT2153-2 | CTGTGGGTGGAAGGCTTCTT | amplicon for NGFG_02153 (*norB*) transcript quantification |
| qRT2042-1 | ATTTTGTCCGAGATGGTTGC | amplicon for NGFG_02042 (*ilvB*) transcript quantification |
| qRT2042-2 | GATAATTTCGCTGCCGTTGT | amplicon for NGFG_02042 (*ilvB*) transcript quantification |
| qRT863-1 | GTGTGATTTTGTGCCGTGAC | amplicon for NGFG_00863 (*glyA*) transcript quantification |
| qRT863-2 | CGCTTCTTTAAACGCTACGG | amplicon for NGFG_00863 (*glyA*) transcript quantification |
|  |  |  |
| NBS162 | AATCAAGCTGCATCAAGCAACTCAA | Northern blot probe for NgncR_162 |
| NBS163 | TATTAACTGACTACTCGAACCAGCT | Northern blot probe for NgncR_163 |

1. Sequences introduced for cloning purposes are given in lower case letters. Restriction sites are underlined.
