# Supplementary material for: Central Role of Sibling Small RNAs NgncR_162 and NgncR_163 in Main Metabolic Pathways of Neisseria gonorrhoeae
Source: mBio. 2023 Jan 4;14(1):e03093-22. doi: 10.1128/mbio.03093-22 (PMC9973317; doi:10.1128/mbio.03093-22)
Supplement: TABLE S1 [file mbio.03093-22-s0009.docx]

**Table S1: Bacterial strains and plasmids used in this study**

| Strain / plasmid | description | reference |
| --- | --- | --- |
|  |  |  |
| *E. coli* |  |  |
| DH5α | F- gyrA96 (Nalr) recA1 relA1 endA1 thi-1 hsdR17(rk-mk+) glnV44 deoRΔ(lacZYA-argF)U169 [Φ80dΔ(lacZ)M15] | Thermo Fisher Scientific |
| Top10 | F- mcrA Δ( mrr-hsdRMS-mcrBC) Φ80lacZΔM15 Δ lacX74 recA1 araD139 Δ(araleu)7697 galU galK rpsL (StrR) endA1 nupG | Thermo Fisher Scientific |
|  |  |  |
| *N. gonorrhoeae* |  |  |
| MS11 | wild-type *N. gonorrhoeae* | laboratory strain  collection |
| MS11 ΔΔ162/163 | MS11 with sRNA genes NgncR_162 and NgncR_163 substituted by a kanamycin resistance cassette | [19] |
| MS11 ΔΔc162 | MS11 ΔΔ162/163 with sRNA gene NgncR_162 inserted in the *iga-trpB* locus | [19] |
| MS11 ΔΔc163 | MS11 ΔΔ162/163 with sRNA gene NgncR_162 inserted in the *iga-trpB* locus | [19] |
| MS11 ΔΔc162/163 | MS11 ΔΔ162/163 with sRNA genes NgncR_162 and NgncR_163 inserted in the *iga-trpB* locus | [19] |
| MS11 93gfp | MS11 carrying a translational NGFG_00093-*gfp* fusion inserted in the *iga-trpB* locus | this study |
| MS11 ΔΔ93gfp | MS11 ΔΔ162/163 carrying a translational NGFG_00093-*gfp* fusion inserted in the *iga-trpB* locus | this study |
| MS11 249gfp | MS11 carrying a translational NGFG_00249-*gfp* fusion inserted in the *iga-trpB* locus | this study |
| MS11 ΔΔ249gfp | MS11 ΔΔ162/163 carrying a translational NGFG_00249-*gfp* fusion inserted in the *iga-trpB* locus | this study |
| MS11 gcvH-F | MS11 expressing GcvH with a C-terminal 3xFLAG-tag | this study |
| MS11 ΔΔgcvH-F | MS11 ΔΔ162/163 expressing GcvH with a C-terminal 3xFLAG-tag | this study |
| MS11 P*_opa_*45 | MS11 expressing NGFG_00045 under control of a neisserial *opa* promoter | this study |
| MS11 ΔΔP*_opa_*45 | MS11 P*_opa_*45 with sRNA genes NgncR_162 and NgncR_163 substituted by a kanamycin resistance cassette | this study |
| MS11 ΔΔP*_opa_*45c | MS11 ΔΔP*_opa_*45 with sRNA genes NgncR_162 and NgncR_163 inserted in the *iga-trpB* locus | this study |
| MS11 P_45_gfp | MS11 with *gfp* replacing NGFG_00045 | this study |
| MS11 P_45_gfpSD | MS11 P_45_gfp with an artificial RBS introduced into the NGFG_00045 5’-UTR | this study |
| MS11 ΔΔP_45_gfp | MS11 ΔΔ162/163 with *gfp* replacing NGFG_00045 | this study |
| MS11 ΔΔP_45_gfpSD | MS11 ΔΔP_45_gfp with an artificial RBS introduced into the NGFG_00045 5’-UTR | this study |
| MS11 Δ45 | MS11 with the DNA segment encompassing 289 nucleotides from the upstream region and 239 bp from the 5’-end of NGFG_00045 replaced by *ermC* | this study |
| MS11 Δc45 | derivative of MS11 Δ45 with reintegration of the DNA segment encompassing 289 nucleotides from the upstream region and 239 bp from the 5’-end of NGFG_00045 | this study |
| MS11 Δ1564 | MS11 with NGFG_01564 substituted by *ermC* | this study |
| MS11 Δ1721 | MS11 with NGFG_01721 substituted by *aadA1* | this study |
|  |  |  |
| plasmids |  |  |
|  |  |  |
| pSL1180 | cloning vector | [70] |
| pMR68 | complementation plasmid for *N. gonorrhoeae* and *N. meningitidis* | [71] |
| pJV300 | plasmid expressing a nonsense sRNA under control of the P_LlacO_ promoter | [29] |
| pXG10-SF | superfolder *gfp*-based translational fusion plasmid | [28] |
| pXG30-SF | superfolder *gfp*-based translational fusion plasmid for intercistronic fusions | [28] |
| pJV-162 | derivative of pJV300 expressing NgncR_162 | [19] |
| pJV-162m1 | derivative of pJV300 expressing NgncR_162 with a mutated SL2 sequence | [19] |
| pXG-863 | pXG30-SF derivative expressing a translational *glyA*-*gfp* fusion | this study |
| pXG-93 | pXG10-SF derivative expressing a translational NGFG_00093-*gfp* fusion | this study |
| pXG-1937 | pXG10-SF derivative expressing a translational NGFG_01937-*gfp* fusion | this study |
| pXG-249 | pXG10-SF derivative expressing a translational NGFG_00249-*gfp* fusion | this study |
| pXG-1146 | pXG10-SF derivative expressing a translational *dnrN*-*gfp* fusion | this study |
| pXG-1722 | pXG30-SF derivative expressing a translational *dadA*-*gfp* fusion | this study |
